# A DNA-Binding Bromodomain-Containing Protein Interacts with and Reduces Rx1-Mediated Immune Response to Potato Virus X

**DOI:** 10.1016/j.xplc.2020.100086

**Published:** 2020-06-16

**Authors:** Octavina C.A. Sukarta, Philip D. Townsend, Alexander Llewelyn, Christopher H. Dixon, Erik J. Slootweg, Lars-Olof Pålsson, Frank L.W. Takken, Aska Goverse, Martin J. Cann

**Affiliations:** 1 Laboratory of Nematology, Department of Plant Sciences, Wageningen University, 6708 PB Wageningen, The Netherlands; 2 Department of Biosciences, Durham University, South Road, Durham DH1 3LE, UK; 3 Biophysical Sciences Institute, Durham University, South Road, Durham DH1 3LE, UK; 4 Department of Chemistry, Durham University, South Road, Durham DH1 3LE, UK; 5 Molecular Plant Pathology, Swammerdam Institute for Life Sciences, University of Amsterdam, Science Park 904, 1098 XH Amsterdam, The Netherlands

**Keywords:** cellular immune response, DNA-binding protein, host–pathogen interaction, plant biochemistry, plant defense, plant virus

## Abstract

Plant NLR proteins enable the immune system to recognize and respond to pathogen attack. An early consequence of immune activation is transcriptional reprogramming. Some NLRs have been shown to act in the nucleus and interact with transcription factors. The Rx1 NLR protein of potato binds and distorts double-stranded DNA. However, the components of the chromatin-localized Rx1 complex are largely unknown. Here, we report a physical and functional interaction between Rx1 and *Nb*DBCP, a bromodomain-containing chromatin-interacting protein. *Nb*DBCP accumulates in the nucleoplasm and nucleolus, interacts with chromatin, and redistributes Rx1 to the nucleolus in a subpopulation of imaged cells. Rx1 overexpression reduces the interaction between *Nb*DBCP and chromatin. *Nb*DBCP is a negative regulator of Rx1-mediated immune responses to potato virus X (PVX), and this activity requires an intact bromodomain. Previously, Rx1 has been shown to regulate the DNA-binding activity of a Golden2-like transcription factor, *Nb*Glk1. Rx1 and *Nb*DBCP act synergistically to reduce *Nb*Glk1 DNA binding, suggesting a mode of action for *Nb*DBCP’s inhibitory effect on immunity. This study provides new mechanistic insight into the mechanism by which a chromatin-localized NLR complex co-ordinates immune signaling after pathogen perception.

## Teaser

The Rx1 NLR protein of potato binds and distorts double-stranded DNA. This study demonstrates physical and functional interaction between Rx1 and *Nb*DBCP, a bromodomain-containing chromatin-interacting protein. *Nb*DBCP is a negative regulator of Rx1-mediated immune responses to potato virus X and acts in concert with Rx1 to reduce the DNA-binding activity of the immune-activating Golden2-like transcription factor, *Nb*Glk1.

## Introduction

The plant’s innate immune system enables cell-autonomous defense responses upon pathogen perception (Maekawa et al., 2011b; Jones et al., 2016). Plant nucleotide-binding leucine-rich repeat (NLR)-type immune receptors directly or indirectly detect pathogen-produced effector proteins and mediate immune responses to the invading pathogen (Staskawicz et al., 2001; Eitas and Dangl, 2010; Maekawa et al., 2011b). NLR proteins are signal-transduction ATPases with numerous domains and have a multidomain structure that allows them to function as a sensor, switch, and response factor (Leipe et al., 2004; Takken and Tameling, 2009).

Plant NLR immune receptors can be broadly divided into three distinct domains. The N terminus typically consists of either a coiled-coil (CC) or Toll–interleukin 1 receptor domain that is involved in inter- and intramolecular interactions (Maekawa et al., 2011a). The nucleotide-binding (NB) domain, sometimes referred to as the NB, Apaf-1, R-proteins, or CED-4 (NB-ARC) domain, forms the central domain and is proposed to function as a nucleotide-dependent molecular switch in NLR activation (van der Biezen and Jones, 1998; Tranier et al., 2000; Takken et al., 2006; Takken and Tameling, 2009). Finally, the C-terminal leucine-rich repeat domain determines pathogen recognition specificity and maintains the NLR protein in an autoinhibited state in the absence of a pathogen-derived signal. The NB-ARC domains of NLRs are bound to ADP in the off state (Tameling et al., 2006; Maekawa et al., 2011a; Williams et al., 2011). Pathogen recognition by the NLR is hypothesized to activate the nucleotide exchange of ADP for ATP, allowing the NB-ARC domain to adopt an activated state and triggering immune signaling, whereas the hydrolysis of ATP to ADP is proposed to re-establish the inactivated state (Tameling et al., 2006). ATP binding in potato Rx1 has been linked to its *in vitro* activity (Fenyk et al., 2015). Recent elucidation of the cryoelectron microscopic structure of *Arabidopsis* ZAR1 in its autoinhibited and activated states provides structural support for this ADP/ATP exchange model (Wang et al., 2019a, 2019b). The NB-ARC domain, however, may not be a strict ATPase for all plant NLRs because, for example, the NB subdomains of rice Os2g_25 900, maize PSiP, and *Arabidopsis* Rpm1 can sequentially cleave phosphates from the nucleotide to the nucleoside *in vitro*, although the *in vivo* role of this activity has yet to be determined (Fenyk et al., 2012).

Proper NLR function often requires a nucleocytoplasmic distribution in the cell. A subset of NLR proteins, including N, MLA10, and Rx1, has a dynamic nuclear-cytoplasmic distribution, whereas RRS1-R becomes localized to the nucleus, dependent on the PopP2 effector (Deslandes et al., 2003; Burch-Smith et al., 2007; Shen et al., 2007; Caplan et al., 2008; Slootweg et al., 2010). Several other NLRs, including barley MLA1 and MLA10, *Arabidopsis* RPS4 and SNC1, and the tobacco N protein, also show nuclear localization (Shen et al., 2007; Wirthmueller et al., 2007; Zhu et al., 2010; Bai et al., 2012). Nuclear expulsion of Rx1, MLA10, N, RPS4, and SNC1 compromises immune activation, suggesting that NLR-dependent signaling components reside in the nucleus (Burch-Smith et al., 2007; Shen et al., 2007; Wirthmueller et al., 2007; Cheng et al., 2009). The identity of these signaling components is therefore of considerable interest.

*In vitro* biochemistry demonstrates that at least a subset of plant NLRs are directly active at DNA. Significant structural homology was proposed between the NLR NB-ARC domain and the DNA replication origin-binding Cdc6/Orc1 proteins (Tameling et al., 2006). In line with this observation, direct interaction with DNA has been observed *in vitro* for potato Rx1, tomato I-2, and the maize orphan NLR PSiP (Fenyk et al., 2015, 2016). The *Rx1* gene, introgressed into potato from the wild species *Solanum tuberosum* ssp. *andigena*, confers resistance to potato virus X (PVX) upon recognition of its coat protein (Bendahmane et al., 1995, 1999). The Rx1 protein binds to genomic DNA *in situ* on immune activation (Fenyk et al., 2015). In addition, Rx1 induces the ATP-dependent bending and melting of DNA *in vitro*. Analysis of Rx1 binding to a variety of DNA structures demonstrated that it favors topologies that resembled transcription bubbles. Rx1 therefore binds, bends, and distorts DNA in a manner reminiscent of the formation of the transcription initiation complex (Finzi and Dunlap, 2010; Liu et al., 2010; Tang et al., 2011; Kim et al., 2012). A further interesting parallel between Rx1 and the eukaryotic Cdc6/Orc1 proteins is that eukaryotic ORCs lack DNA sequence specificity *in vitro* but show a higher affinity for specific DNA topologies (Remus et al., 2004; MacAlpine et al., 2010). Consistent with this, Rx1 showed no observed sequence specificity but did show an increased affinity for branched and melted DNA topologies over linear double-stranded DNA (dsDNA).

One of the most important and earliest consequences of immune activation is transcriptional reprogramming (Navarro et al., 2004; Tsuda et al., 2008; Garner et al., 2016). The associations of MLA10 with Myb and WRKY transcription factors (TFs) and N with the TF SPL6 suggest that plant NLRs themselves may directly regulate transcription during the immune response (Chang et al., 2013; Padmanabhan et al., 2013; Roberts et al., 2013). The specificity of an immune-dependent transcription response is difficult to reconcile with the observation that Rx1 DNA-binding specificity may be mediated by local DNA topology rather than sequence specificity, implying the involvement of other factors in conferring specificity. Indeed, the CC of Rx1 has been shown to interact with a Golden2-like (GLK) TF, *Nb*Glk1 (Townsend et al., 2018). *Nb*Glk1 binds distinct to consensus DNA sequences, and this binding affinity is reduced upon its interaction with Rx1 *in vitro*. Moreover, *Nb*Glk1 overexpression activates immune responses to PVX. Such a direct involvement of GLK-like TFs in defense signaling has also been reported in *Arabidopsis* toward cucumber mosaic virus (Han et al., 2016) and the fungal pathogens *Fusarium graminearum* (Savitch et al., 2007) and *Hyaloperonospora arabidopsidis* (Murmu et al., 2014).

Transcriptional reprogramming initiated as part of an immune response must be under tight control and is likely to be exercised at multiple levels (Garner et al., 2016). Rx1 associates with *Nb*Glk1 and prevents its assembly on DNA unless Rx1 is activated by PVX, representing one level of transcriptional control (Townsend et al., 2018). Among other potential mechanisms, histone modifications are of particular interest because they represent an important mechanism to control the transcription of defense-related genes (Espinas et al., 2016). For example, histone modification through the removal of acetyl groups from modified lysine residues by histone deacetylases can suppress immunity (Ding et al., 2012; Wang et al., 2017).

Here, we set out to identify nuclear regulators of Rx1 function and investigate their role in immunity. We report that Rx1 interacts directly with a DNA-binding bromodomain (BD)-containing protein (*Nb*DBCP), thereby identifying a new member of the nuclear DNA bound NLR complexes that control plant immunity. The finding that *Nb*DBCP acts as a negative regulator of Rx1-mediated immune responses to PVX provides a direct link between chromatin and immunity.

## Results

### The CC Domain of Rx1 Interacts with a BD-Containing Protein

**Figure 1:**
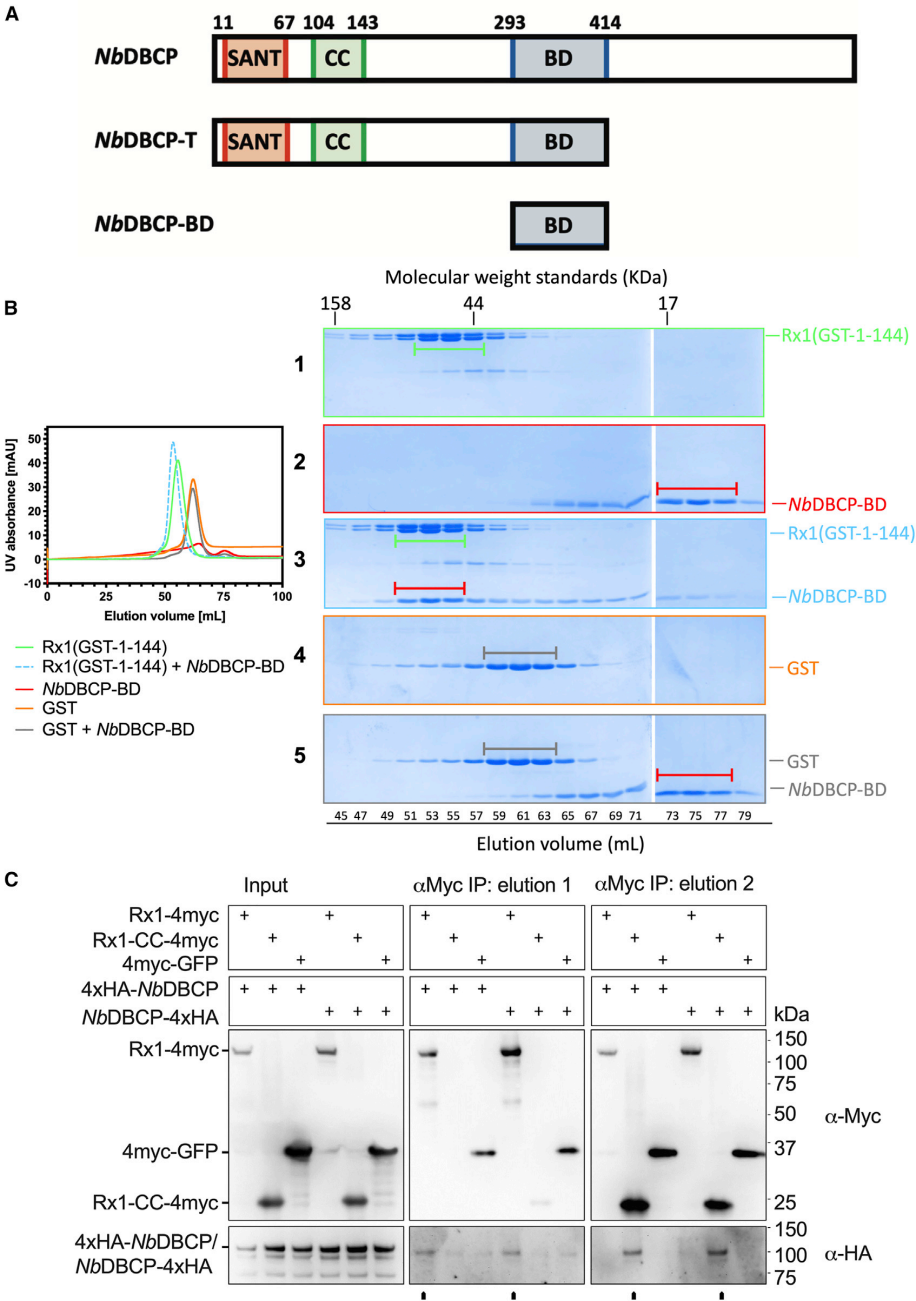
Rx1 Binds *Nb*DBCP *In Vitro* and *In Planta*. **(A)** Top: diagram of the *Nb*DBCP domain structure. Middle and bottom: *Nb*DBCP-T and *Nb*DBCP-BD represent proteins used in this study. Numbers represent amino acid residues. SANT, DNA-binding SANT-type helix-turn-helix domain. CC, coiled-coil domain; BD, bromodomain. **(B)** Interaction of Rx1(GST-1–144) with *Nb*DBCP-BD. On the left are representative gel filtration chromatograms of Rx1(GST-1–144), *Nb*DBCP-BD, GST, and Rx1(GST-1–144) incubated with *Nb*DBCP-BD, and GST incubated with *Nb*DBCP-BD. Peak fractions were visualized by SDS–PAGE and are represented by capped bars. **(C)** Co-immunoprecipitation of 4xMyc-tagged full-length Rx1 (Rx1-4xMyc) or Rx1-CC (Rx1-CC-4xMyc) when co-expressed *in planta* with N- and C-terminally 4xHA-tagged *Nb*DBCP (4xHA-*Nb*DBCP and *Nb*DBCP-4xHA). The labels on the figure are as follows: input denotes the constructs agroinfiltrated into *N*. *benthamiana* leaves; αMyc, an immunoblot performed using an anti-myc epitope tag antibody; αHA, an immunoblot performed using an anti-HA epitope tag antibody; αMyc IP, immunoprecipitation of the denoted input samples with an anti-myc epitope tag antibody; elution indicates the method used to release immunoprecipitated protein (see Supplemental Information). Immunoblot bands corresponding to Rx1-4xMyc, Rx1-CC-4xMyc, 4xMyc-GFP, and *Nb*DBCP from the αMyc and αHA immunoblots are indicated. See also Supplemental Figure 1.

To provide further insight into Rx1’s mechanism of action and its control of the *Nb*Glk1 TF, we screened for additional Rx1 interactors. *Nb*Glk1 was previously identified in a yeast two-hybrid (Y2H) screen as an interactor of the CC domain of Rx1 (amino acids 1–144). Here, we used the same CC domain to perform an additional Y2H screen of a random-primed *Nicotiana benthamiana* mixed tissue cDNA library. Niben101Scf17137g00006.1 (https://solgenomics.net) was identified in seven clones corresponding to four individual cDNAs, two of which were isolated twice. Individual clones were presumably obtained multiple times due to the amplification of the random-primed cDNA library. The full-length cDNA for Niben101Scf17137g00006.1 encodes a protein of 664 amino acids with a predicted molecular weight of 74 814 Da. The protein carries a single Swi3, Ada2, N-Cor, and TFIIIB (SANT)-type helix-turn-helix domain and a single BD of 111 amino acids (Figure 1A). We therefore named it *N*. *benthamiana* DNA-binding BD-containing protein (*Nb*DBCP). We used the Simple Modular Architecture Research Tool (Letunic and Bork, 2018) to identify all proteins that had a similar domain structure consisting of a SANT-type domain and a BD. Proteins with domain structures similar to *Nb*DBCP were identified as uncharacterized proteins in both dicots (e.g., At2g44430 of *Arabidopsis thaliana*) and monocots (e.g., LOC4346003 of *Oryza sativa* Japonica Group). A maximum likelihood phylogenetic analysis demonstrated that the proteins formed two distinct clades, with branches for each clade receiving strong support with bootstrap values of 0.98 (Supplemental Figure 1). Of the 57 plant proteins that had domain structures similar to *Nb*DBCP, 43 (including *Nb*DBCP) also carried a CC domain between the SANT-type domain and the BD. *Nb*DBCP is therefore representative of a larger protein family with a conserved domain structure.

### Rx1 Interacts Directly with *Nb*DBCP *In Vitro* and *In Vivo*

A BD typically recognizes acetylated lysine residues. Since all *Nb*DBCP clones identified from the Y2H screen encompassed the BD, Rx1 probably interacts with this domain. To test this hypothesis, we assessed the binding of Rx1 to the BD domain of *Nb*DBCP (referred to as *Nb*DBCP-BD hereafter) *in vitro*. *Nb*DBCP-BD is encoded by amino acids 293–414 of the *Nb*DBCP ORF (Figure 1A). We therefore expressed amino acids 293–414 of *Nb*DBCP (*Nb*DBCP-BD) as a recombinant protein and examined its interaction with a glutathione-S-transferase (GST)-tagged Rx1-CC domain (Rx1(GST-1–144)) by size-exclusion chromatography. The extreme C terminus of Rx1(GST-1–144) was susceptible to some proteolytic cleavage during purification, causing the purified protein to run as a doublet in SDS–PAGE. We noted shifts in the peak bands that corresponded to Rx1(GST-1–144) (Figure 1B, SDS–PAGE panel 1, capped green bar) and *Nb*DBCP-BD (Figure 1B, SDS–PAGE panel 2, capped red bar) when they were co-incubated (Figure 1B, panel 3). By contrast, GST alone (Figure 1B, SDS–PAGE panel 4) showed no peak shift when co-incubated with *Nb*DBCP-BD (Figure 1B, SDS–PAGE panel 5). These results suggest that *Nb*DBCP-BD and Rx1-CC interact *in vitro*.

We then examined whether full-length *Nb*DBCP interacts with full-length Rx1 *in planta*. Co-immunoprecipitation experiments were performed using full-length *Nb*DBCP fused to an *N*- or *C*-terminal 4xHA epitope tag (Figure 1C; 4xHA-*Nb*DBCP and *Nb*DBCP-4xHA) with full-length Rx1 fused to a 4xMyc epitope tag (Figure 1C; Rx1-4xMyc). Experiments were also performed with the Rx1 CC domain fused to a 4xMyc epitope tag (Figure 1C; Rx1-CC-4xMyc). GFP fused to a 4xMyc epitope tag (Figure 1C; 4xMyc-GFP) served a control for non-specific interactions with *Nb*DBCP. Two different elution protocols were used to optimize the recovery of potential complexes formed between *Nb*DBCP and Rx1-4xMyc (Figure 1C; elution 1) or Rx1-CC-4xMyc (Figure 1C; elution 2). Both the N- and C-terminal 4xHA *Nb*DBCP fusions could be detected with an anti-HA tag antibody after immunoprecipitation with an anti-myc antibody when they were co-expressed with Rx1-CC-4xMyc (Figure 1C; elution 2) or Rx1-4xMyc (Figure 1C; elution 1). The amount of N- and C-terminally tagged *Nb*DBCP that co-immunoprecipitated with the full-length Rx1-4xMyc was less than that with Rx1-CC-4xMyc. Nevertheless, the amount of N- and C-terminally tagged *Nb*DBCP that co-immunoprecipitated with the full-length Rx1 construct was consistently and repeatedly above background levels. Elution 1 did not liberate Rx1-CC-4xMyc (Figure 1C; elution 1; αMyc panel), whereas elution 2 did release Rx1-4xMyc (Figure 1C; elution 2; αMyc panel). However, the αHA western blot was optimized to observe interactions between *Nb*DBCP and Rx1-CC-4xMyc and was not exposed for a sufficient length of time to observe interactions between *Nb*DBCP and Rx1-4xMyc. Why less *Nb*DBCP was immunoprecipitated with the full-length Rx1 molecule compared with the CC domain is unknown. Nonetheless, the interaction between the full-length *Nb*DBCP and Rx1 *in planta* was observed across independent experiments and thus represents a genuine interaction.

### Rx1 and *Nb*DBCP Localize to the Nucleus and Nucleolus *In Situ*

**Figure 2:**
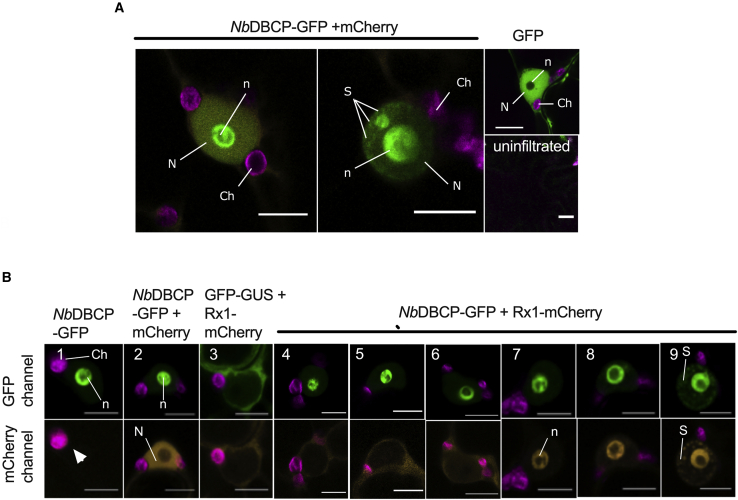
The Distribution of *Nb*DBCP in Cells. **(A)** Subcellular distribution of *Nb*DBCP-GFP *in planta*. Representative overlay confocal images of *N*. *benthamiana* leaf epidermal cells transiently expressing *Nb*DBCP-GFP + P19 (left and middle panels) or free GFP (upper right panel), or an uninfiltrated leaf (lower right panel). Images were taken at 2 dpi. Scale bars, 10 μm. N, nucleus; n, nucleolus; S, subnuclear bodies; Ch, chloroplasts. **(B)** Subcellular distribution of *Nb*DBCP-GFP with or without mCherry or Rx1-mCherry. The P19 silencing suppressor was included for all combinations. Images were taken at 2 dpi. Scale bars, 10 μm. White arrow indicates the bleed-through of GFP in the mCherry channel as observed in the nucleus. Similar settings were used for all images, including 488 and 543 nm laser intensities (3% and 55%–56.5%, respectively). (1) *Nb*DBCP-GFP expressed alone. (2) *Nb*DBCP-GFP expressed with mCherry. (3) GFP-GUS expressed with Rx1-mCherry. (4–9) *Nb*DBCP-GFP expressed with Rx1-mCherry. N, nucleus; n, nucleolus; S, subnuclear bodies; Ch, chloroplasts. See also Supplemental Figures 2–5.

Having established that Rx1 interacts with *Nb*DBCP both *in vitro* and *in planta*, we set out to identify the intracellular localization of this event. We used confocal laser scanning microscopy to examine the cellular localization of a C-terminal fusion of *Nb*DBCP with GFP (*Nb*DBCP-GFP) in *N*. *benthamiana* epidermal cells in the absence and presence of Rx1 (Rx1-mCherry). The subcellular distribution of Rx1-mCherry in the cytoplasm and nucleus was within the range reported in existing studies (Supplemental Figure 2) (Slootweg et al., 2010; Tameling et al., 2010). *Nb*DBCP-GFP in combination with free mCherry (Figure 2A; mCh) is localized in the nucleoplasm and nucleolus (Figure 2A; left and middle panels; Supplemental Figure 3). These localization patterns do not vary substantially between 2 and 3 dpi or in the presence or absence of the P19 silencing suppressor, minimizing the likelihood that the nucleolar distribution of *Nb*DBCP-GFP is an artifact of overexpression (Supplemental Figure 3). The amount of nucleoplasmic signal varied between cells (compare Figure 2A with the GFP channel of Figure 2B, panels 4–9), and in a few cells, *Nb*DBCP-GFP aggregated in subnuclear bodies (Figure 2A; image at lower right panel taken at 3 dpi). No signal was observed in uninfiltrated cells. The distribution of *Nb*DBCP-GFP differed from that of free GFP, confirming that the localization of *Nb*DBCP-GFP is genuine (Figure 2A; upper and lower right panels).

The nuclear localization pattern of *Nb*DBCP-GFP was unaltered when it was co-expressed with Rx1-mCherry (Figure 2B; GFP channel; compare panel 1 with panels 4–9). In addition, the cellular distribution of Rx1-mCherry in the nucleus relative to the cytoplasm was mostly unaffected and similar to that observed upon co-expression with GFP-GUS (Figure 2B; mCherry channel; compare panel 3 with panels 4–6). We confirmed this result by determining the intensity ratios of the mCherry signal in the nucleus relative to the cytoplasm (I_N_/I_C_), as described previously (Slootweg et al., 2010). The I_N_/I_C_ ratios of Rx1-mCherry did not differ significantly in the presence or absence of *Nb*DBCP-GFP (Supplemental Figure 2). However, in some cells, we observed that Rx1-mCherry relocalized to the nucleolus and to subnuclear bodies when it was co-expressed with *Nb*DBCP-GFP (23% ± 7% of imaged cells (SD; 51 cells imaged in four biologically independent experiments); Figure 2B; mCherry channel; compare panels 4–6 with panels 7–9). Rx1-mCherry was present in the cytoplasm and nuclear envelope when it was not relocalized to the nucleolus (Figure 2B, panels 4–6). The weaker signal of mCherry was due to a lower quantum yield compared with GFP, and the apparent brighter signal of Rx1 in the nucleolus (Figure 2B, panels 7–9) likely reflected a high local concentration. In addition, the Rx1-mCherry construct used in this study is intact (as demonstrated by immunoblotting in Supplemental Figure 4) but expressed at significantly lower levels than free mCherry.

Given that only a low proportion of cells showed the redistribution of Rx1-mCherry into the nucleolus, we quantified this shift in all imaged cells. To do so, the relative intensity ratios of Rx1-mCherry in the nucleolus relative to the nucleoplasm (I_Nucleolus_/I_Nucleoplasm_) were determined. Data from independent experiments indicated that there was a statistically significant increase in nucleolar signal (Supplemental Figure 5). Notably, the possibility of an overexpression artifact still remains. To explore this, we demonstrated that *Nb*DBCP-GFP exhibited very low levels of bleed-through in the mCherry channel when expressed alone (Figure 2B, panel 1; note the lack of signal in the mCherry channel compared with the GFP channel). The observed redistribution of Rx1-mCherry upon co-expression with *Nb*DBCP-GFP far exceeds this background level (Figure 2B; mCherry channel; panels 2–9). In addition, this redistribution was not observed when Rx1-mCherry was expressed with GFP-GUS (Figure 2B; mCherry channel; panel 3; Supplemental Figure 5). Together, these observations minimize the likelihood that the accumulation of Rx1 into subnuclear bodies is due to experimental artifacts. In summary, the observed shared subcellular distribution of Rx1 and *Nb*DBCP in the nucleus (and the nucleolus in a subset of cells) is consistent with their ability to interact.

### Rx1 and *Nb*DBCP Interact with DNA *In Situ*

The co-localization of Rx1 and *Nb*DBCP to the nucleus prompted us to investigate whether Rx1 and *Nb*DBCP interact at DNA *in planta*.

**Figure 3:**
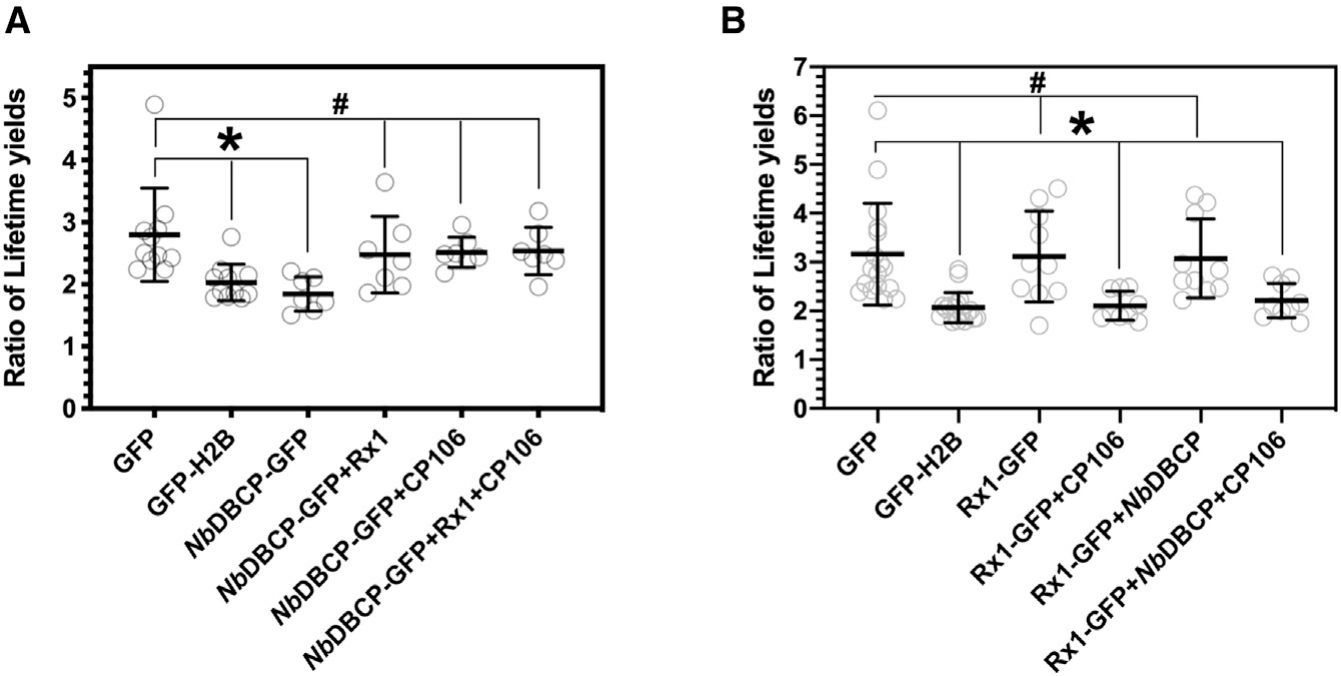
Binding of Rx1 and *Nb*DBCP Proteins to Chromatin *In Situ.* **(A)** The ratio of the long to short GFP lifetimes for the *Nb*DBCP-GFP full-length construct alone and upon co-expression with Rx1 and the avirulent CP106 allele of the PVX CP (scatterplot ± SD; ∗*p* < 0.05, #*p* > 0.05; one-way ANOVA with post hoc Dunnett’s multiple comparison). **(B)** The ratio of the long to short GFP lifetimes for the Rx1-GFP full-length construct alone and upon co-expression with *Nb*DBCP and the avirulent CP106 allele of the PVX CP (scatterplot ± SD; ∗*p* < 0.05, #*p* > 0.05; one-way ANOVA with post hoc Dunnett’s multiple comparison). See also Supplemental Figure 6.

We studied Rx1-*Nb*DBCP-DNA interactions using a Förster resonance energy transfer-fluorescence lifetime imaging microscopy (FRET-FLIM). GFP (negative control), histone H2B fused to GFP (GFP-H2B; positive control), full-length Rx1 with or without an N-terminal GFP tag, or full-length *Nb*DBCP with or without an N-terminal GFP tag were transiently expressed in *N*. *benthamiana*. The constituent fluorescence lifetimes for the GFP tag were examined in leaves counterstained with the nucleic acid stain LDS 751 (Figure 3). GFP showed two distinct fluorescence lifetimes at ∼0.5 and ∼1.5 ns. The fluorescence lifetime at ∼0.5 ns can be explained by energy transfer from GFP to an acceptor fluorophore (Fenyk et al., 2015). Increased energy transfer from GFP to acceptor LDS 751 (indicative of physical proximity to nucleic acids) increases the relative contribution of the ∼0.5-ns fluorescent lifetime. A shift in the ratio of the ∼1.5 (long)- to ∼0.5 (short)-ns GFP lifetimes is indicative of interactions with DNA (Fenyk et al., 2015) at distances within the Förster radius (likely <50 Å). We monitored the interaction of an *Nb*DBCP-GFP fusion with DNA with or without Rx1 (untagged) in the presence or absence of the Rx1-activating PVX coat protein CP106. This experiment would reveal whether *Nb*DBCP interacts with chromatin *in situ* and whether this interaction is altered by the co-expression of Rx1 and CP106. Consistent with the localization of *Nb*DBCP to the nucleus (Figure 2), *Nb*DBCP-GFP bound DNA when expressed alone (Figure 3A) However, *Nb*DBCP did not bind DNA when it was co-expressed with either Rx1 or CP106, despite an equivalent fluorescent signal indicating that *Nb*DBCP was still expressed. The ability of CP106 alone to displace *Nb*DBCP from DNA prompted us to ask whether CP106 and *Nb*DBCP can physically interact. CP106 did not interact with *Nb*DBCP in a Y2H assay (Supplemental Figure 6), suggesting that the effect observed on *Nb*DBCP DNA binding is due to an indirect interaction *in planta*. While we cannot formally exclude such an interaction, there are currently no data to support it. Future experiments using a time course of co-immunoprecipitation and bifluorescence complementation experiments on the co-expression of CP106 and *Nb*DBCP *in planta* will help to resolve whether this is a true negative result.

Next, we monitored the interaction of an Rx1-GFP fusion with chromatin with or without *Nb*DBCP (untagged) and in the presence or absence of the Rx1-activating (avirulent) PVX coat protein (CP106) (Figure 3B). In line with our previous studies, Rx1-GFP expressed without *Nb*DBCP only bound DNA in the presence of CP106 (Fenyk et al., 2012). The Rx1-GFP fusion did not interact with chromatin when it was co-expressed with *Nb*DBCP (untagged) (Figure 3B). This result is consistent with the observation that *Nb*DBCP-GFP did not interact with chromatin when it was co-expressed with Rx1 (untagged) (Figure 3A). Interestingly, when Rx1-GFP was co-expressed with *Nb*DBCP and CP106, it was observed to interact with chromatin (Figure 3B).

### *Nb*DBCP Reduces Rx1-Mediated Immune Responses

The observed interaction between *Nb*DBCP and Rx1 suggests a functional relationship between these proteins. Rx1 can trigger two distinct types of immune outputs: (1) a cell death response that can be induced by overexpression of the PVX coat protein, and (2) a symptomless extreme resistance response that results in the inhibition of viral replication (Bendahmane et al., 1999).

Virus-induced gene silencing (VIGS) uses a recombinant TRV virus that carries part of a designated plant gene sequence that is targeted for silencing (Lange et al., 2013). We used VIGS to silence the endogenous *Nb*DBCP-encoding gene of *N*. *benthamiana* to investigate whether it is required for Rx1-mediated immune responses. Two independent VIGS constructs were designed to target different regions of the *Nb*DBCP transcript to ensure that the results were not due to off-target effects.

**Figure 4:**
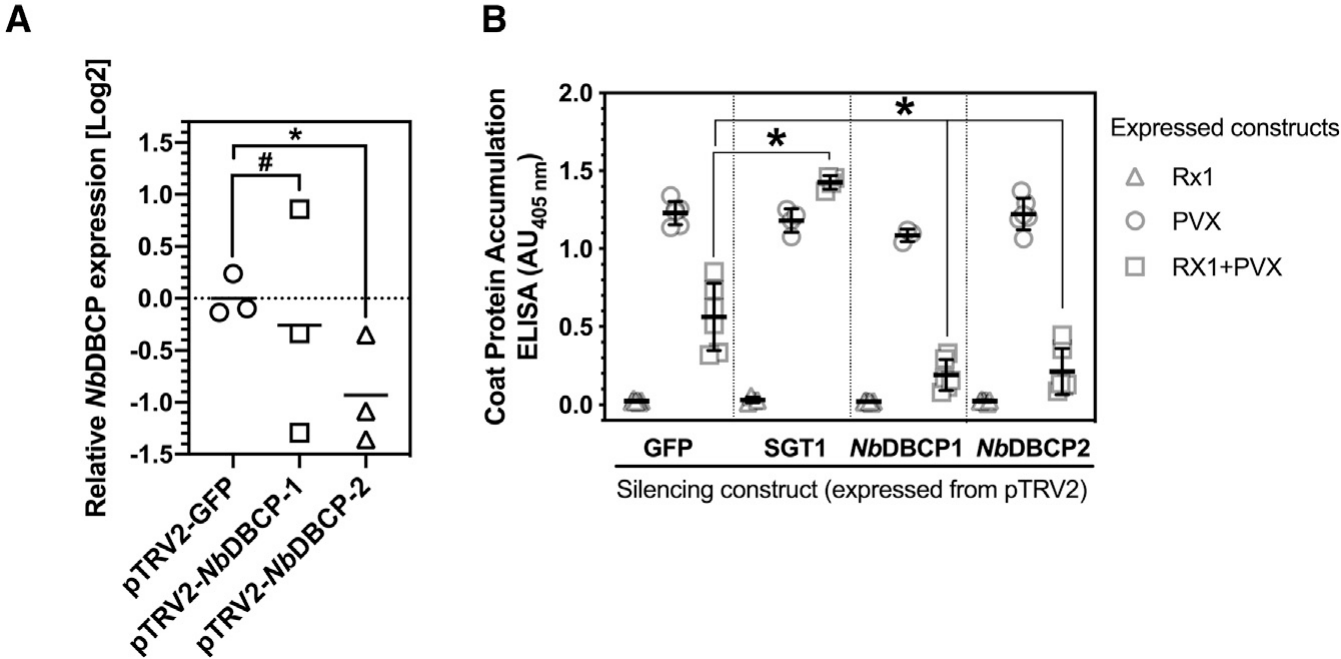
The Influence of *Nb*DBCP Gene Silencing on Susceptibility to PVX. **(A)** The relative expression level of *Nb*DBCP in *N*. *benthamiana* TRV-VIGS plants as determined by qRT–PCR analysis. Each data point represents a biological replicate consisting of pooled leaf materials from at least three different plants, and three technical replicates. Significance level is calculated based on the log2 transformation of 2−ΔΔCt using a paired Student’s *t*-test with ⍺ = 0.05 (scatterplot ± SD; ∗*p* < 0.05, #*p* > 0.05). The crossbar designates the mean relative *Nb*DBCP expression. **(B)** Absorbance at 405 nm, indicative of PVX coat protein accumulation, in four VIGS strains (pTRV2-GFP [negative control], pTRV2-SGT1 [positive control], pTRV2-*Nb*DBCP-1, and pTRV2-*Nb*DBCP-2) when co-infiltrated with Rx1 and PVX (scatterplot ± SD; *n=*3–6; ∗*p* < 0.05 compared with pTRV2-GFP Rx1 + PVX; one-way ANOVA with Dunnett’s multiple comparison). The data were derived from six independent experiments for pTRV2-GFP, pTRV2-*Nb*DBCP-1, and pTRV2-*Nb*DBCP-2 and four independent experiments for pTRV2-SGT1.

The downregulation of *Nb*DBCP mRNA by VIGS was examined by real-time qPCR (Figure 4A). Rx1-mediated immunity was assessed by measuring PVX coat protein accumulation following agroinfiltration using an infectious PVX clone. A VIGS construct targeting GFP served as a negative control for Rx1-mediated immune responses. No change in PVX coat protein accumulation (in the absence of Rx1) was observed in *Nb*DBCP-silenced plants (pTRV2-*Nb*DBCP-1 and -2) compared with *GFP*-silenced plants (pTRV2-GFP; Figure 4B). Therefore, *Nb*DBCP alone does not influence PVX accumulation.

As expected, PVX coat protein accumulation was reduced when Rx1 was co-expressed with PVX in either non-silenced or *GFP*-silenced plants (pTRV2-GFP; Figure 4B). A TRV::SGT1 silencing construct was used as a positive control for compromised Rx1 function. SGT1 is a homolog of a yeast ubiquitin ligase-associated protein; it has a general role in NLR-mediated immunity and is required for Rx1 function (Peart et al., 2002; Lu et al., 2003; Slootweg et al., 2010; Hoser et al., 2014). PVX coat protein accumulation was unaffected in *SGT1*-silenced plants (pTRV2-SGT1) in the absence of Rx1. However, in the presence of Rx1, SGT1 silencing eliminated immunity, resulting in increased accumulation of PVX coat protein (Figure 4B). In addition, PVX coat protein accumulation was significantly reduced when Rx1 and PVX were co-expressed in *Nb*DBCP-silenced plants (pTRV2-*Nb*DBCP-1 and -2) compared with *GFP*-silenced plants (pTRV2-GFP) (Figure 4B). These findings suggest that *Nb*DBCP suppresses Rx1-mediated resistance response. Our qRT–PCR analysis demonstrated a trend of decreased relative *Nb*DBCP expression in pTRV2:*Nb*DBCP-1 and -2 compared with pTRV2:GFP plants. Despite the minor effect and variability in gene silencing (see Figure 4A, pTRV2:*Nb*DBCP-1), the findings of our disease assays were robust and replicable across experiments (Figure 4). This may indicate that Rx1-mediated immunity is sensitive to a relatively small decrease in *Nb*DBCP expression. Overall, these data suggest that *Nb*DBCP is a negative regulator of Rx1-mediated immunity.

### *Nb*DBCP-BD Affects Immune Responses to PVX

*Nb*DBCP-BD is sufficient for the interaction between Rx1 and *Nb*DBCP (Figure 1B). We therefore investigated whether the BD domain was crucial to *Nb*DBCP-mediated immunity to PVX.

**Figure 5:**
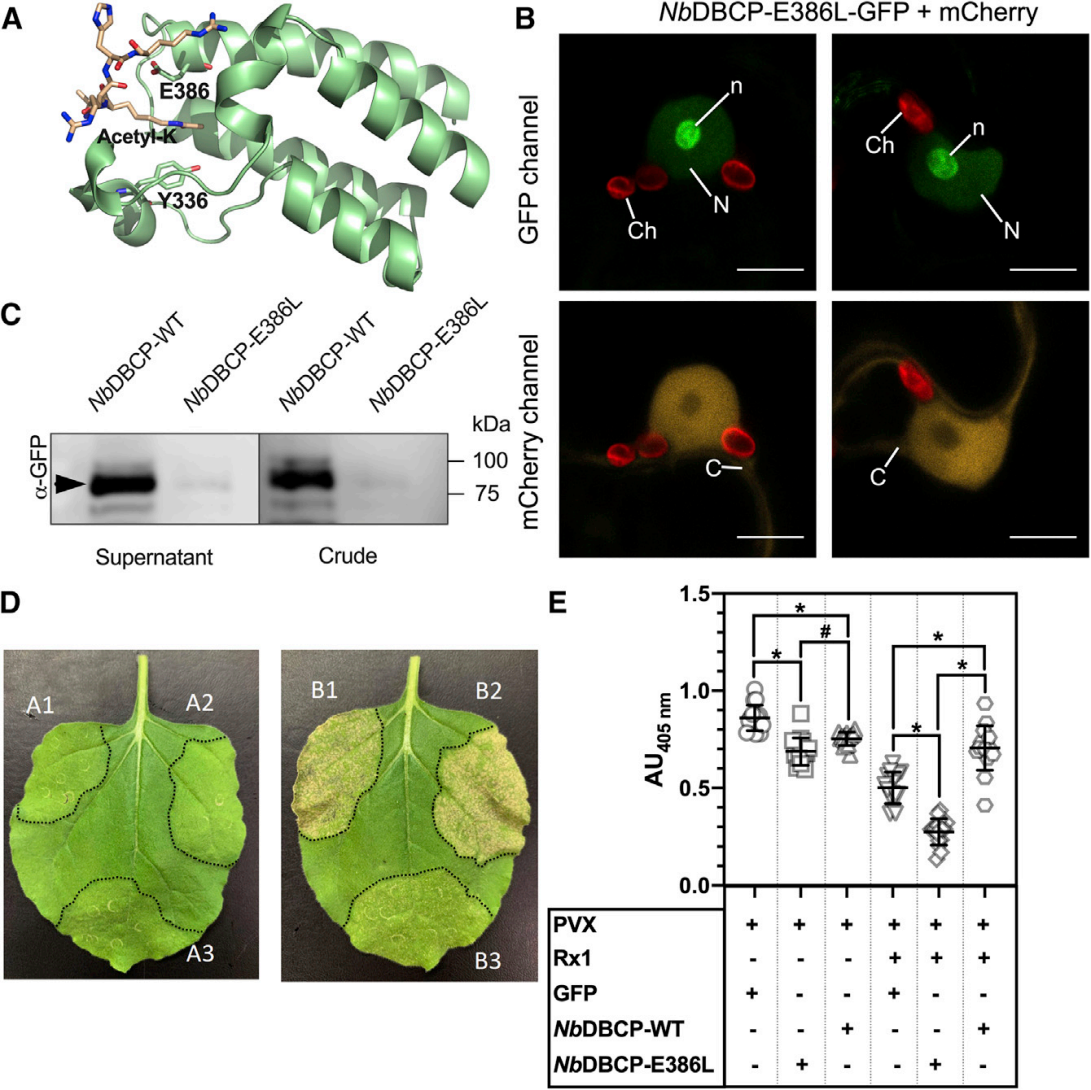
The Influence of *Nb*DBCP-BD Mutation on Rx1-mediated Reductions in Susceptibility to PVX. **(A)** BD residues Y336 and E386 are shown, as is the acetyl-lysine of a modeled target protein. **(B)** Representative images of the nuclei of *N*. *benthamiana* leaf epidermal cells co-expressing *Nb*DBCP-E386L-GFP and free mCherry. Images were taken at 2 dpi by confocal microscopy with consistent results in all imaged cells. Scale bars, 10 μm. N, nucleus; n, nucleolus; C, cytoplasm; Ch, chloroplast. **(C)** Western blot of the *Nb*DBCP-WT-GFP and *NbDBCP-GFP-*E386L protein expression + P19 *in planta*. α-GFP-immunoblot performed using an anti-GFP antibody. Equal protein loading was assessed with a Coomassie Blue protein loading control. Leaf samples harvested at 3 dpi were used for protein extraction. kDa, molecular weight markers. **(D)** Representative photographs of *N*. *benthamiana* leaves infiltrated with pGR208 (which drives the expression of a PVX amplicon) and/or full-length Rx1 in the presence/absence of *Nb*DBCP-WT or the *NbDBCP-*E386L variant. Images were taken at 5 dpi with consistent results among leaf samples. A1, pGR208 + GFP; A2, pGR208 + *Nb*DBCP-WT; A3, pGR208 + *Nb*DBCP-E386L; B1, pGR208 + Rx1 + GFP; B2, pGR208 + Rx1 + *Nb*DBCP-WT; B3, pGR208 + Rx1 + *Nb*DBCP-E386L. **(E)** Chart representing relative PVX levels measured by DAS–ELISA in *N*. *benthamiana* leaves infiltrated with pGR208 and/or Rx1 in the presence or absence of *Nb*DBCP-WT or *Nb*DBCP-E386L. Leaves were harvested at 5 dpi. Error bars represent the SD (means ± SD; *n* = 16; ∗*p* < 0.05, #*p* > 0.05; one-way ANOVA with post hoc Tukey multiple comparison test). See also Supplemental Figures 7 and 8.

*Nb*DBCP-BD was analyzed using the Phyre^2^ protein fold recognition engine and modeled using the BPTF (Bromodomain and PHD finger-containing transcription factor) BD in complex with histone H4 acetylated at Lys16 (PDB: 3QZT). Y336 and E386 in *NbDBCP* were identified as candidate residues that interact with acetyl-lysine and therefore required for BD-dependent function (Figure 5A). To study their effect on immune responses to PVX, *Nb*DBCP-Y336F and *Nb*DBCP-E386L mutants were generated, conserving amino acid side-chain bulk while ablating intermolecular interactions at the site. Unfortunately, the *Nb*DBCP-Y336F variant could not be expressed *in planta* and was excluded from further studies.

The cellular localization of a C-terminal fusion of *Nb*DBCP-E386L with GFP (*Nb*DBCP-E386L-GFP) in *N*. *benthamiana* epidermal cells was determined using confocal laser scanning microscopy. *Nb*DBCP-E386L-GFP was co-expressed with the P19 silencing suppressor to enhance expression, and the fusion protein was found to localize to the nucleoplasm and nucleolus (Figure 5B; GFP channel), similar to that observed for the *Nb*DBCP-WT-GFP protein.

*Nb*DBCP-E386L expression *in planta* was compared with that of the wild-type *Nb*DBCP by western blot. The *NbDBCP*-E386L protein was detectable but its expression level was consistently lower than that of *Nb*DBCP-WT (Figure 5C). This prevented us from further assessing whether and how E386L affected the suppressive effect of *Nb*DBCP on Rx1-mediated immune responses by studying the loss-of-function phenotype, as it can be explained by reduced *Nb*DBCP-E386L protein levels. We therefore investigated whether *Nb*DBCP-E386L confers a possible gain-of-function phenotype *in planta*, as such a phenotype is less likely a result of decreased protein expression. We investigated the effect of overexpression wild-type and mutant *Nb*DBCP (*Nb*DBCP-GFP-E386L) on phenotypes induced by PVX. *Nb*DBCP and PVX were transiently expressed in *N*. *benthamiana* upon *A*. *tumefaciens* infiltration. As expected, no immune response was observed when PVX was co-expressed with GFP (Figure 5D and Supplemental Figure 7; A1), *Nb*DBCP-GFP-WT (Figure 5D and Supplemental Figure 7; A2), or *Nb*DBCP-GFP-E386L (Figure 5D and Supplemental Figure 7; A3) in the absence of Rx1. However, a hypersensitive response was observed when PVX and GFP were co-expressed with Rx1 (Figure 5D and Supplemental Figure 7; B1) and when PVX and *Nb*DBCP-GFP-WT were co-expressed with Rx1 (Figure 5D and Supplemental Figure 7; B2). Interestingly, the levels of cell death were qualitatively reduced when PVX and *Nb*DBCP-GFP-E386L were co-expressed with Rx1 (Figure 5D and Supplemental Figure 7; B3). The gain-of-function phenotype for *Nb*DBCP-E386L cannot be explained by a failure to interact with Rx1, as both the *Nb*DBCP-WT and *Nb*DBCP-E386L interact with the CC domain of Rx1 in a Y2H assay (Supplemental Figure 8).

To further validate these data, we directly measured virus coat protein accumulation in infiltrated *N*. *benthamiana* leaves to provide quantitative support for the qualitative analysis of Figure 5D. Virus coat protein accumulation in leaf infiltrates was measured by double antibody sandwich ELISA (DAS–ELISA) using an antibody that recognizes the PVX coat protein. In the absence of Rx1, both the *Nb*DBCP-WT and *Nb*DBCP-E386L proteins increased the basal immune response of *N*. *benthamiana* cells (Figure 5E; compare the second and third datasets with the first), which is evident through a decrease in the accumulation of PVX coat protein. Consistent with our hypothesis, Rx1 co-expression indeed significantly reduced PVX virus expression (Figure 5E; compare the first and fourth datasets). Notably, more viruses accumulated when Rx1 was co-expressed with *Nb*DBCP-WT-GFP than with GFP (Figure 5E; compare the fourth and sixth datasets). This supports the observation from VIGS that *Nb*DBCP-WT suppresses Rx1 activity. Significantly, less viral accumulation was observed when Rx1 was co-expressed with *Nb*DBCP-E386L-GFP (Figure 5E; compare fourth and fifth datasets). The significant increase in Rx1-mediated immunity can therefore be attributed to a gain-of-function phenotype conferred by the *Nb*DBCP-E386L-GFP variant, demonstrating that *Nb*DBCP is a negative regulator of Rx1 function.

### *Nb*DBCP Reduces *Nb*Glk1-Mediated DNA Binding

We hypothesized that *Nb*DBCP might inhibit the pro-immune activity of Rx1 as part of a larger complex. *Nb*Glk1 binds to consensus GLK DNA binding sites, and its binding affinity for these sites is reduced by Rx1 (Townsend et al., 2018). Because Rx1 is a negative regulator of *Nb*Glk1 DNA binding, we investigated whether *Nb*DBCP reduces Rx1-mediated immunity via affecting *Nb*Glk1 DNA binding.

**Figure 6:**
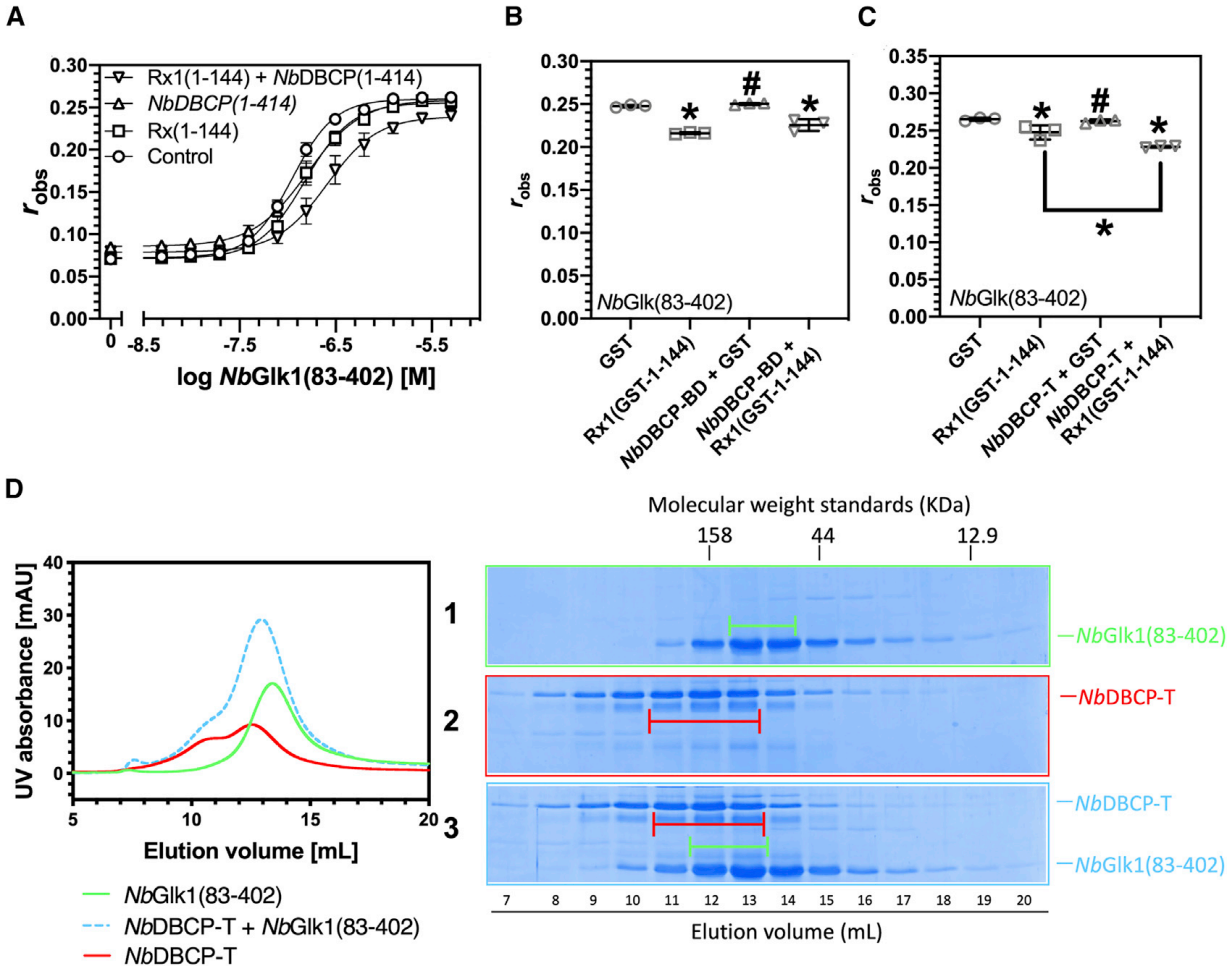
Interactions between *Nb*DBCP, Rx1, and *Nb*Glk1 *In Vitro*. **(A)** The influence of Rx1 and *Nb*DBCP on *Nb*Glk1 DNA binding. Fluorescence anisotropy values plotted against log protein concentration for *Nb*Glk1(83–402) in the presence or absence of Rx1(1–144) or *Nb*DBCP-T (*n* = 3). **(B)** DNA binding of *Nb*Glk1(83–402) was measured by fluorescence anisotropy in the presence or absence of GST, Rx1(GST-1–144), and *Nb*DBCP-BD (means ± SEM; *n* = 3; ∗*p* < 0.05, #*p* > 0.05; one-way ANOVA with post hoc Tukey multiple comparison test). **(C)** DNA binding of *Nb*Glk1(83–402) was measured by fluorescence anisotropy in the presence or absence of GST, Rx1(GST-1–144), and *Nb*DBCP-T (means ± SEM; *n* = 3; ∗*p* < 0.05, #*p* > 0.05; one-way ANOVA with post hoc Tukey multiple comparison test). **(D)** Interaction of *Nb*Glk1(83–402) with *Nb*DBCP-T. On the left are representative gel filtration chromatograms of *Nb*Glk1(83–402), *Nb*DBCP-T, and *Nb*Glk1(83–402) incubated with *Nb*DBCP-T. Peak fractions were visualized by SDS–PAGE and are represented by capped bars. See also Supplemental Figure 9.

We were unable to express the full-length *Nb*DBCP molecule as a recombinant protein *in vitro*. However, we were able to express a C-terminally truncated variant of *Nb*DBCP that consisted of amino acids 1–414 (*Nb*DBCP-T). We measured the K_d_ value of *Nb*Glk1 for a dsDNA substrate that contained a concatenated GGATATCC *Nb*Glk1 binding site (Townsend et al., 2018) in the presence or absence of Rx1(GST-1–144) and/or *Nb*DBCP-T by fluorescence anisotropy (Figure 6A). Rx1(GST-1–144) reduced the binding affinity of *Nb*Glk1 for its dsDNA substrate, as expected, from 0.11 ± 0.00 μM (SD) to 0.15 ± 0.01 μM (SD). Interestingly, *Nb*DBCP-T also reduced the binding affinity of *Nb*Glk1 for its dsDNA substrate to 0.16 ± 0.01 μM (SD). In addition, Rx1(GST-1–144) and *Nb*DBCP-T exhibited a synergistic effect, leading to a much greater reduction in the binding affinity of *Nb*Glk1 for its dsDNA substrate to 0.26 ± 0.03 μM (SD).

As the interaction between Rx1 *Nb*DBCP is BD-dependent, we investigated whether *Nb*DBCP-BD alone was sufficient to reduce the binding affinity of *Nb*Glk1 for its dsDNA substrate. *Nb*DBCP-BD did not alter *Nb*Glk1 binding to dsDNA in either the presence or absence of Rx1, indicating that the site of the Rx1-*Nb*DBCP interaction is not required for the influence of *Nb*DBCP on *Nb*Glk1 DNA binding (Figure 6B). The experiment of Figure 6B was performed using *Nb*DBCP-T instead of *Nb*DBCP-BD (Figure 6C). *Nb*DBCP-T reduced *Nb*Glk1 binding to dsDNA in the presence of Rx1, supporting the findings of Figure 6A and confirming that the failure of *Nb*DBCP-BD to reduce *Nb*Glk1 DNA binding was not specific to the assay conditions.

The ability of Rx1 and *Nb*DBCP to synergistically occlude *Nb*Glk1 DNA binding suggests that they are able to form a larger protein complex. We therefore investigated whether *Nb*Glk1 and *Nb*DBCP form a complex *in vitro*. We expressed amino acids 1–414 of *Nb*DBCP (*Nb*DBCP-T) as a recombinant protein and examined its interaction with amino acids 83–402 of *Nb*Glk1 (*Nb*Glk1(83–402)) by size-exclusion chromatography. Both *Nb*DBCP-T and *Nb*Glk1(83–402) represent the largest recombinant protein variants we have been able to produce as soluble proteins in *E*. *coli*. We noted a shift in the peak band corresponding to *Nb*Glk1(83-402) (Figure 6D, SDS–PAGE panel 1, capped green bar) when it was co-incubated with *Nb*DBCP-T (Figure 6D, panel 3, capped green bar), which was indicative of complex formation. No shift in the peak band corresponding to *Nb*DBCP-T (Figure 6D, SDS–PAGE panel 2, capped red bar) was obvious when it was incubated with *Nb*Glk1(83–402) (Figure 6D, panel 3, capped red bar). However, a more sensitive quantitative examination of the distribution of *Nb*DBCP protein to the left- (higher molecular weight) and right-hand sides of the protein peak at an elution volume of 12 mL by densitometry revealed an enhanced distribution of *Nb*DBCP-T toward a higher molecular weight. The left:right ratio of *Nb*DBCP within the 12-mL elution volume was 1.37 for *Nb*DBCP-T alone but 1.60 when *Nb*DBCP-T was incubated with *Nb*Glk1(83–402), indicative of a shift due to complex formation.

The interaction between *Nb*Glk1 and *Nb*DBCP observed through fluorescence anisotropy was therefore demonstrated through gel filtration analysis. However, gel filtration analysis indicated that the interaction is relatively transient and only weakly detectable on the transit of the complex through a gel filtration column. Accordingly, we were unable to observe a three-way interaction between Rx1, *Nb*DBCP, and *Nb*Glk1 by gel filtration chromatography.

We performed additional confocal imaging to investigate the influence of *Nb*DBCP overexpression on the cellular localization of *Nb*GLK1-GFP. Experiments were performed using an HA-tagged version of *Nb*DBCP because an *Nb*DBCP-mCherry construct was not available. Immunoblotting demonstrated that all proteins used for imaging were expressed and intact (Supplemental Figure 9A). The data demonstrated that *Nb*DBCP-HA overexpression does not alter the subcellular distribution of *Nb*GLK1-GFP (Supplemental Figure 9B). This does not preclude the possibility of a transient interaction between the two proteins.

In conclusion, *Nb*DBCP is an immune regulator that acts on an Rx1 and *Nb*Glk1 complex at chromatin, and its ability to modulate Rx1 activity is dependent on intact BD.

## Discussion

Here, we identify the BD-containing protein *Nb*DBCP as an Rx1-interacting protein (Figure 1). At least one *Nb*DBCP-like protein is predicted within the genome sequences of a range of dicot and monocot species (Supplemental Figure 1). *Nb*DBCP-like proteins are therefore widespread in higher plants, but their functions are unknown. The localization of *Nb*DBCP to the nucleolus suggests a broadly conserved function in plant ribosome biogenesis that awaits further investigation. The identification of *Nb*DBCP provides a potential direct link between histone modification and NLR activity. Although antibodies that detect endogenous *Nb*DBCP were not available, (Figure 4A), we can infer its relative expression levels by comparison with actin in TRV:GFP plants based on qPCR analysis. Data from independent repeats demonstrate that the average Ct values between actin and *Nb*DCBP are within 1 Ct difference. This indicates that the expression levels of endogenous *Nb*DBCP are comparable to those of actin.

*Nb*DBCP was shown to localize to the nucleolus (Figure 2), and approximately a quarter of the cells showed the redistribution of Rx1 to the nucleolus upon *NbDBCP and Rx1 co-expression* (Figure 2). However, the reasons for this localization is unknown. We note that the nucleolus is a key target for plant viruses (Ding and Lozano-Duran, 2020; Kalinina et al., 2018), and nucleolar chromatin is subject to histone modification (Saez-Vasquez and Delseny, 2019). Therefore, it is possible that the localization of Rx1 and *Nb*DBCP to the nucleolus functions as part of sub-organellar specific defense response, but this awaits future investigation. Whether Rx1 and *Nb*DBCP are further redistributed upon PVX infection is an open question. When expressed alone, *Nb*DBCP-GFP interacts with plant chromatin *in situ* (Figure 3A), and its DNA binding *in situ* appears to be inhibited by either Rx1 or CP106 overexpression. It is interesting to note that Rx1-GFP does interact with chromatin when co-expressed with *Nb*DBCP and CP106 (Figure 3B). This suggests that CP106, or the encoded mRNA, rearranges a complex at chromatin that results in the loss of an *Nb*DBCP–chromatin interaction but permits the interaction of Rx1 with chromatin. The FRET-FLIM experiment measures a ratio of long to short GFP lifetimes, and so this observation is not an artifact of protein expression. This surprising finding suggests that CP106, or its mRNA, can affect *Nb*DBCP activity in an Rx1-independent manner. The PVX coat protein has been proposed to interact with multiple host proteins in *N*. *benthamiana* (Park et al., 2009), and their identities remain a question for future research. Our interpretation of the confocal microscopy and FRET-FLIM data is that *Nb*DBCP localizes in the nucleolus where it interacts—or is closely associated—with chromatin. Such an interaction is consistent with the known role of BDs to bind acetyl-lysine that is typically found in histones (Dhalluin et al., 1999; Marmorstein and Berger, 2001). These latter interactions can be disturbed upon the overexpression of Rx1 or the CP, possibly via a third protein that interacts with all partners. However, a caveat for the interpretation of the FRET-FLIM data is the possibility of a false-negative result. If the expressed *Nb*DBCP-GFP fusion protein has saturated all available DNA-binding sites, the accumulation of an increased pool of non-DNA-bound protein will shift the ratio of the long to short lifetimes to the GFP negative control. In the absence of an available alternative method that is not susceptible to the same issue of false negatives, however, the interpretation of a negative result should be viewed with some caution. A conservative interpretation of the data is therefore that *Nb*DBCP is able to interact with DNA *in situ*, with some evidence that either Rx1 or CP106 redistributes *Nb*DBCP from this site. It is interesting to note that a similar observation was made previously for the interaction of *Nb*Glk1 with DNA *in situ* (Townsend et al., 2018). Rx1 redistributed *Nb*Glk1 from DNA *in situ* and reduced its binding affinity for DNA *in vitro*.

We were unable to demonstrate an interaction between *Nb*DBCP-BD and small molecules N-acetyl-lysine and ϖ-acetyl-histamine (which mimics the N-acetyl-lysine side chain) by isothermal calorimetry. However, the substrate specificity of BD for acetylated proteins is also dependent on binding interactions with the amino acids that surround the acetylated lysine (Mujtaba et al., 2002). The binding affinity for the acetylated lysine is therefore possibly too low to observe in the absence of an appropriate peptide sequence. An alternative possibility is that the BD shows specificity for a different chromatin modification. For example, the typical YN motif of the BD L_BC_ loop is replaced by YF in *NbDBCP*. The bulky phenylalanine may therefore clash with N-acetyl-lysine, as observed previously for BDs with alternative residues at this site (Wen et al., 2014).

We used an indirect approach to determine whether histone modification binding was required for *Nb*DBCP function. Using the structural modeling of *Nb*DBCP-BD, we identified two residues (Y336 and E386) that were possibly involved in *Nb*DBCP-BD interactions with a modified target protein (Figure 5A). Only the *Nb*DBCP-E386L variant could be expressed *in planta*, but it accumulated at levels below that of the wild-type protein. However, upon its accumulation, the *Nb*DBCP-E386L variant showed a gain-of-function phenotype that resulted in a potentiated Rx1-mediated immune response to PVX compared with the wild-type protein (Figure 5D and 5E). Because it involves a gain-of-function phenotype, the finding is genuine despite the reduced expression level of the mutant protein compared with the wild-type protein. This finding is consistent with the VIGS data of Figure 4B, demonstrating that *Nb*DBCP is a negative regulator of Rx1-mediated immunity. Extreme resistance and cell death are thought to constitute distinct pathways of Rx1 immunity (Bendahmane et al., 1999). The common view is that cell death occurs as a secondary resistance response when extreme resistance proves insufficient. As cell death was qualitatively more prominent, our data suggest that *Nb*DBCP wild-type expression reduces extreme resistance (consistent with the gene-silencing data of Figure 4B), whereas *Nb*DBCP-E386L expression enhances extreme resistance (due to less cell death). The precise mechanism of the *NbDBCP-E386L* gain-of-function phenotype is not known. *NbDBCP-E386L* may, for example, form non-functional complexes with the wild-type protein, causing a net reduction in the negative effect on Rx1 responses. This mechanism awaits the future investigation of whether wild-type *Nb*DBCP co-expression can rescue the phenotype associated with *Nb*DBCP overexpression.

Rx1 and *Nb*DBCP acted synergistically to reduce the affinity of *Nb*Glk1 for dsDNA (Figure 6). The DNA-binding assay used had no histones present, and so the observed effect on *Nb*Glk1 unlikely involved binding to chromatin modification. This is further demonstrated by the observation that isolated *Nb*DBCP-BD does not influence *Nb*Glk1 DNA binding (Figure 6). It is most likely, therefore, that Rx1 and *Nb*DBCP act in a complex to physically occlude *Nb*Glk1 from DNA. We have several lines of evidence that *Nb*DBCP and *Nb*Glk1 are able to form a complex (Figure 6). It is interesting to note that the *Nb*DBCP-T protein had an approximate molecular weight consistent with the formation of a dimer, as assessed by gel filtration chromatography (Figure 6D). The functional relevance of a higher-order structure for *Nb*DBCP-T is unknown. Unfortunately, *Nb*DBCP-T-E386L could not be produced as a recombinant protein in *E*. *coli* to investigate whether the altered formation of such a higher-order structure could explain the mutant phenotype and to investigate the relevance of such a structure. A crucial direction for future work, therefore, will be to fully define this larger Rx1-*Nb*Glk1-*NB*DBCP complex (and potential homo-oligomerization) *in planta* and determine the requirements for its formation and complete role in immunity.

**Figure 7:**
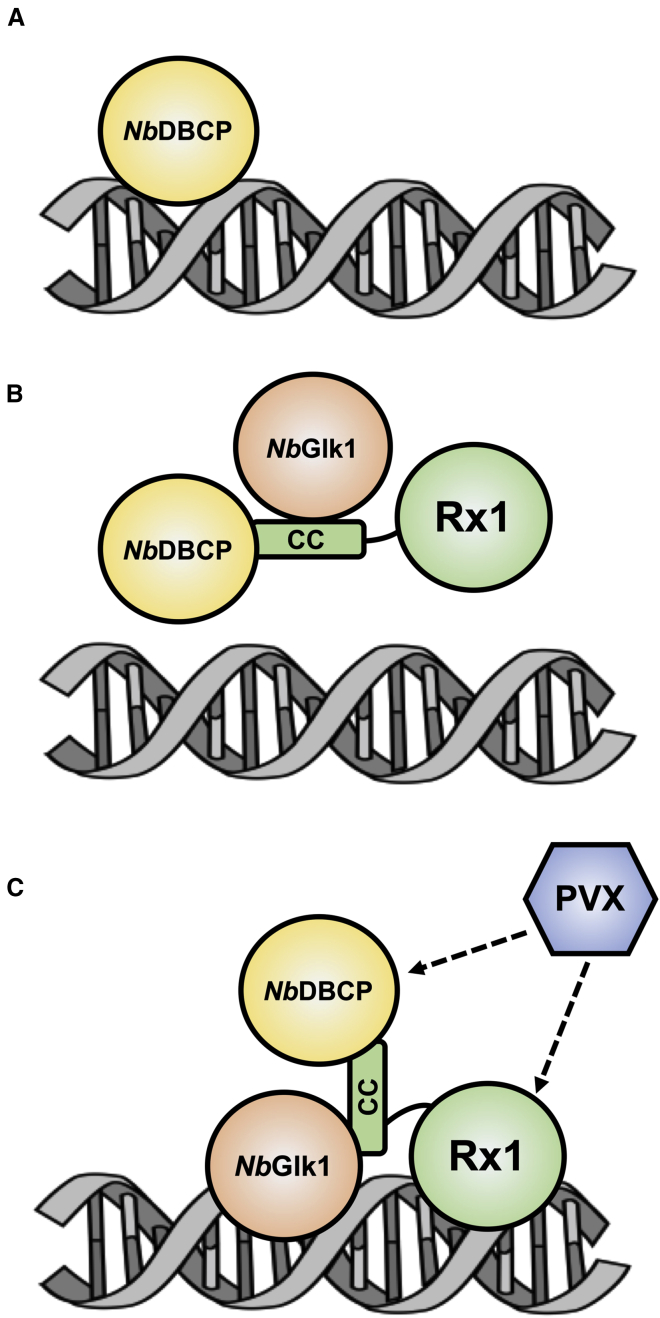
Model for Protein Interactions with Rx1. **(A)***Nb*DBCP interacts with DNA. **(B)** In the absence of PVX, Rx1 interacts with *Nb*DBCP and separately with *Nb*Glk1 at DNA and reduces the affinity of *Nb*Glk1 for DNA (Townsend et al., 2018). *Nb*DBCP suppresses immunity downstream of the Rx1/*Nb*Glk1-DNA interaction. **(C)** PVX acts via Rx1 and/or *Nb*DBCP, resulting in a complex where Rx1 but not *Nb*DBCP interacts with chromatin. Instead, *Nb*Glk1 interacts with chromatin (Townsend et al., 2018). The dotted line indicates where direct interaction is not identified.

A possible interpretation of the data presented here and elsewhere (Townsend et al., 2018) centers on DNA. *Nb*DBCP interacts with DNA (Figures 3A and 7A), presumably at the nucleolus (Figure 2), and suppresses immune responses associated with Rx1 through an unknown mechanism that likely depends on a functional BD (Figures 4 and 5). Co-expressed Rx1 and *Nb*Glk1 form an inactive complex at DNA (Townsend et al., 2018). Co-expressed Rx1 and *Nb*DBCP also form a complex *in planta* (Figure 1C). When Rx1 and *Nb*DBCP are co-expressed, neither interact with DNA (Figures 3 and 7B). However, the nature of the DNA contacts formed by Rx1, *Nb*Glk1, and *Nb*DBCP at endogenous levels in the cell (in the absence of PVX) is not fully resolved. The model shown in Figure 7B should therefore be viewed with some caution, as the multi-protein complex and its interactions with DNA may well be dynamic and/or transient. Size-exclusion chromatography (Figure 6D) suggests that the multi-protein complex formed between Rx1, *Nb*Glk1, and *Nb*DBCP (Figure 7B) is possible, albeit transient, in the absence of PVX, although proof of its existence *in planta* awaits further experiment. The immune activation of Rx1 and/or *Nb*DBCP via PVX CP, whether direct or indirect, permits an uncharacterized change in the relative orientation of Rx1, *Nb*Glk1, and *Nb*DBCP with respect to DNA (Figure 7C). Rx1 and *Nb*Glk1 interact with DNA in the presence of PVX when co-expressed (Townsend et al., 2018). *Nb*DBCP does not interact with DNA in the presence of PVX when co-expressed, whereas Rx1 does (Figures 3 and 7C). These data are consistent with the orientation of molecules with respect to DNA that is shown in Figure 7C. However, the exact nature of the multi-protein complex and its composition when Rx1, *Nb*Glk1, *NB*DBCP, and PVX CP are all present may be different from that shown and awaits further investigation. Immune signaling is likely to be activated when *Nb*Glk1 is stably bound to its consensus sequences (Townsend et al., 2018) and *Nb*DBCP is removed from its inhibitory site (Figure 3A).

In conclusion, we identify *Nb*DBCP as an immune-suppressing protein that acts at chromatin and is regulated by Rx1. Rx1 provides a direct link between PVX perception and transcriptional processes at DNA. Furthermore, the CC domain appears to function as a complex scaffold for nuclear proteins involved in transcriptional reprogramming. This immune-regulated complex at chromatin is regulated by nuclear-localized Rx1 to suppress immune activation until the perception of an appropriate pathogen signal.

## Methods Summary

All plasmids were produced using standard molecular biology techniques. Proteins for *in vitro* analysis were produced as recombinants in *E*. *coli* and purified by affinity chromatography. Y2H analysis was performed by Hybrigenics Services SAS (Paris, France). Protein interaction studies were performed by gel filtration chromatography or immunoprecipitation after expression *in planta*. The protein-DNA binding was analyzed by fluorescence anisotropy. Protein subcellular distribution studies were performed by laser scanning confocal microscopy. Gene knockdown *in planta* was performed by VIGS. Protein expression in plants was performed by *Agrobacterium tumefaciens*-mediated agroinfiltration. Several differences with the computed open reading frame for Niben101Scf17137g00006.1 were noted. The cloned *Nb*DBCP open reading frame is deposited at NCBI under accession number MN594539. Full methods details are provided in the Supplemental Information.

## Funding

This work was supported by 10.13039/501100000268Biotechnology and Biological Sciences Research Council grant BB/M007405/1 (to M.J.C. and L.-O.P.), the 10.13039/501100003958Dutch Technology Foundation STW and Earth and Life Sciences ALW (to E.J.S., O.C.A.S., and A.G.), and VICI project no. 865.14.003 (to F.L.W.T.) (Netherlands Organization for Scientific Research).

## Author Contributions

Conceptualization, M.J.C. and A.G.; Methodology, M.J.C., A.G., P.D.T., O.C.A.S., C.H.D., E.J.S., and L.-O.P.; Investigation, P.D.T., O.C.A.S., C.H.D., and E.J.S.; Writing – Original Draft, M.J.C.; Writing – Review & Editing, M.J.C., A.G., F.L.W.T., and O.C.A.S.; Funding Acquisition, M.J.C., L.-O.P., A.G., E.J.S., and F.L.W.T.; Resources, A.L. and F.L.W.T.; Supervision, M.J.C., A.G., and L.-O.P.

## Supplemental Information

Document S1. Supplemental Methods and Supplemental Figures 1–9

Document S2. Article plus Supplemental Information

## Footnotes

Supplemental Information is available at *Plant Communications Online.*
