# Supplementary material for: A DNA-Binding Bromodomain-Containing Protein Interacts with and Reduces Rx1-Mediated Immune Response to Potato Virus X
Source: Plant Commun. 2020 Jun 16;1(4):100086. doi: 10.1016/j.xplc.2020.100086 (PMC7371201; doi:10.1016/j.xplc.2020.100086)
Supplement: Document S1. Supplemental Methods and Supplemental Figures 1–9 [file mmc1.pdf]

Plant Communications, Volume *1*

Supplemental Information

A DNA-Binding Bromodomain-Containing Protein Interacts with and Reduces Rx1-Mediated Immune Response to Potato Virus X

Octavina C.A. Sukarta, Philip D. Townsend, Alexander Llewelyn, Christopher H. Dixon, Erik J. Slootweg, Lars-Olof Pålsson, Frank L.W. Takken, Aska Goverse, and Martin J. Cann

SUPPLEMENTAL METHODS

Oligonucleotides – All oligonucleotide sequences used for this study are provided in the Supplemental Table.

Plasmids - The *Nb*DBCP (Niben101Scf17137g00006.1 (https://solgenomics.net) open-reading frame was amplified by PCR from cDNA synthesized from *N. benthamiana* whole leaf material and the DNA was sequenced on both strands using Sanger DNA sequencing. Several differences with the computed open-reading frame for Niben101Scf17137g00006.1 were noted. The cloned open-reading frame is deposited at NCBI with Accession number MN594539. A PCR product spanning *Nb*DBCP residues 293-414 was cloned into the XhoI site of pET14b (pET14b- *Nb*DBCP-BD) and fitted with a hexahistidine tag for affinity purification of recombinant protein. The oligonucleotides used to construct pET14b- *Nb*DBCP-BD were *Nb*DBCP-1 and *Nb*DBCP-2. A PCR product spanning *Nb*DBCP residues 1-414 was cloned into the XhoI site of pET14b (pET14b- *Nb*DBCP-T) and fitted with a hexahistidine tag for affinity purification of recombinant protein The oligonucleotides used to construct pET14b- *Nb*DBCP-BD were *Nb*DBCP-7 and *Nb*DBCP-8.

PCR products spanning nucleotides 1-300 (*Nb*DBCP-1) and 645-945 (*Nb*DBCP-2) of the *Nb*DBCP coding sequence were cloned into the NcoI and XhoI sites of pTRV2 (pTRV2-*Nb*DBCP-1 and pTRV2-*Nb*DBCP-2). pTRV2-SGT1 is as previously described (Gabriels et al., 2006; Gabriels et al., 2007).

A PCR product encompassing the full-length *Nb*DBCP cDNA was cloned into the NcoI and NotI sites of pRAP35S-YFP-4HA to make pRAP35S-*Nb*DBCP-4HA. The oligonucleotides used to construct pRAP35S-*Nb*DBCP-4HA were *Nb*DBCP-3 and *Nb*DBCP-4. An AscI/PacI fragment from pRAP35S-*Nb*DBCP-4HA encompassing the 35S promoter and *Nb*DBCP-4HA fusion was cloned into the corresponding sites of the binary vector pBIN+ to make pBIN35S-*Nb*DBCP-4HA. A PCR product encompassing the full-length native *Nb*DBCP cDNA was introduced into Gateway donor vector pDONR207 (Invitrogen) to make pDONR207-*Nb*DBCP. The *Nb*DBCP gene was then recombined into the Gateway destination binary vector pK7WGF2 (Karimi et al., 2002) to make GFP-*Nb*DBCP. The oligonucleotides used to make pDONR207-*Nb*DBCP were *Nb*DBCP-5 and *Nb*DBCP-6.

Mutant constructs were generated by site directed mutagenesis. The oligonucleotides used to make *Nb*DBCP E386L variants were *Nb*DBCP-9 and *Nb*DBCP-10. The oligonucleotides used to make *Nb*DBCP Y336F variants were *Nb*DBCP-11 and *Nb*DBCP-12.

Plasmids pET14b-*Nb*Glk1(83-402), pET22b-Rx1-CCNBARC, pGEX-6P-1-Rx1(GST-1-144) and pBIN35S-*Nb*Glk1-4HA are as described (Townsend et al., 2017). pBIN-35S-based plasmids corresponding to Rx1, GFP-H2B, Rx1-GFP, Rx1-mCherry, Rx1-4myc and CP106 are as described (Townsend et al., 2017). The PVX:GFP construct used were pGr106 (Townsend et al., 2018) and pGr208 which is based on pGr106 and modified to express GFP5 (Haseloff et al., 1997) driven by a duplicated PVX coat protein promoter. For TRV-VIGS silencing, the pTRV1 construct was used as described (Liu et al., (2002).

Protein Expression and Purification - Protein corresponding to Rx1-CCNBARC was expressed from pET22b-Rx1-CCNBARC as described (Townsend et al., 2017). Rx1 CC domain fused to GST (Rx1(GST-1-144) was expressed from pGEX-6P-1-Rx1(GST-1-144) as described (Townsend et al., 2017). Protein corresponding to *Nb*Glk1(83-402) was expressed from pET14b-*Nb*Glk1(83-402) as described (Townsend et al., 2017).

Protein corresponding to *Nb*DBCP-BD wild type and *Nb*DBCP-BD-Y336F was expressed from pET14b-*Nb*DBCP-BD and pET14b-*Nb*DBCP-BD-Y336F plasmids in *E. coli* BL21(DE3) pLysS. A starter culture was grown overnight at 37°C in Luria broth supplemented with 50 μg mL^{-1} kanamycin and 34 μg mL^{-1} chloramphenicol. The overnight culture was diluted 1:50 into fresh Luria broth with antibiotics and grown with shaking at 37°C to $A_{600\,nm}$ = 0.8. Protein production was induced at 37°C with 1 mM isopropyl-β-D-thiogalactoside for 2 hours. Cells were centrifuged (4,000 g, 20 minutes, 4°C). Pelleted cells were washed with 50 mM Tris-HCl pH 8.5, 1 mM EDTA and centrifuged (5,500 g, 20 min, 4°C). Cells were resuspended in twice their volume of lysis buffer (50 mM Tris-HCl pH 8.0, 200 mM NaCl, 40 mM imidazole, 5 mM β-mercaptoethanol, and SIGMAFAST™ Protease Inhibitor Cocktail Tablets). Cells were lysed by sonication (150 s) and the lysate cleared by centrifugation at (42,000 g, 60 minutes, 4°C). The supernatant was loaded onto a 5 mL HisPrep HP Ni-NTA column (GE Healthcare) on an AKTA Pure chromatography system at 2 mL min^{-1} (GE Healthcare). The column was washed with 5 bed volumes of lysis buffer, 20 bed volumes of wash buffer (lysis buffer + 1 M NaCl), 5 bed volumes of lysis buffer, and eluted with lysis buffer supplemented with 500 mM imidazole. Peak fractions were assessed by SDS-PAGE, pooled, concentrated, exchanged into storage buffer (50 mM Tris-HCl pH 7.5, 500 mM NaCl, 1 mM EDTA, 1 mM DTT, 20 % (v/v) glycerol), and stored at -80°C.

Protein corresponding to *Nb*DBCP-T was expressed from pET14b-*Nb*DBCP-T plasmid in *E. coli* BL21(DE3). A starter culture was grown overnight at 37°C in Luria broth supplemented with 50 μg mL^{-1} kanamycin. The overnight culture was diluted 1:50 into fresh Luria broth with antibiotics and grown with shaking

at 37°C to $A_{600\ nm}$ = 0.6. Protein production was induced at 22°C with 0.5 mM isopropyl-β-D-thiogalactoside for 18 hours. Cells were centrifuged (4,000 g, 20 minutes, 4°C). Pelleted cells were washed with 50 mM Tris-HCl pH 8.5, 1 mM EDTA and centrifuged (5,500 g, 20 min, 4°C). Cells were resuspended in twice their volume of lysis buffer (50 mM Na_2PO_4 pH7.5, 200 mM NaCl, 1 mM DTT and SIGMAFAST™ Protease Inhibitor Cocktail Tablets). Cells were lysed by sonication (150 s) and the lysate cleared by centrifugation at (42,000 g, 60 minutes, 4°C). The supernatant was loaded onto a 5 mL HisPrep HP Ni-NTA column (GE Healthcare) on an AKTA Pure chromatography system at 2 mL min^{-1} (GE Healthcare). The column was washed with 5 bed volumes of lysis buffer, 20 bed volumes of wash buffer (lysis buffer + 500 mM NaCl), 5 bed volumes of lysis buffer, and eluted with lysis buffer supplemented with 200 mM imidazole. Peak fractions from the Ni-NTA column were pooled, concentrated and loaded onto a Superdex 200 16/600 and eluted with 50 mM Na_2PO_4 pH 7.5, 200 mM NaCl, 1 mM DTT, 1 mM EDTA). Peak fractions were assessed by SDS-PAGE, pooled, concentrated, exchanged into storage buffer (50 mM Tris-HCl pH 7.5, 500 mM NaCl, 1 mM EDTA, 1 mM DTT, 20 % (v/v) glycerol), and stored at -80°C.

Yeast Two-Hybrid Analyses - Hybrigenics Services SAS (Paris, France) performed the yeast two-hybrid screen using Rx1 (amino acids 1-144) cloned into pB27 bait plasmid as a C-terminal fusion to LexA (N-LexA-Rx1-C). The screen was performed against a random-primed *N. benthamiana* mixed tissue cDNA library constructed into pP6 prey plasmid. A total of 96.6 million clones (approximately 9-fold library coverage) were screened following a mating approach with Y187 (*MATα*) and L40 Gal4 (*MATα*) yeast strains as described (Fromont-Racine et al., 1997). To confirm protein-protein interactions, freshly transformed yeast colonies were resuspended in 1 mL sterile deionized water, and 10 μL aliquots were spotted onto medium lacking leucine and tryptophan (−L/−W) and medium lacking leucine, tryptophan, histidine (−L/−W/−H), supplemented with 10 or 50 mM 3-Amino-1,2,4-triazole (3-AT). Growth was scored after 5 to 7 d of incubation at 28°C. CP106 (amino acids 1-237) was cloned into pB66 bait plasmid as a C-terminal fusion to Gal4 (N-Gal4-CP106-C).

Gel Filtration Analysis - Gel filtration analysis of protein was performed at 4°C using an Sephacryl HiPrep 16/60 S200 HR column (GE Healthcare) on an AKTA Pure chromatography system (GE Healthcare). Protein was dialysed overnight against running buffer (20 mM Tris-HCl pH 7.4, 140 mM NaCl, 1 mM DTT). Proteins were incubated on ice for 30 min individually or together and then centrifuged at 12,000 g for 30 min. Protein loading

concentration was approximately 75 μM. Columns were run at a flow rate of 0.5 mL/min in running buffer. Thirty μL of each eluted fraction was subjected to SDS-polyacrylamide gel electrophoresis (SDS-PAGE) and visualization with Quick Coomassie stain (Generon). Molecular weights were calibrated with gel filtration standards (BioRad): bovine thyroglobulin (670 kDa), bovine γ-globulin (158 kDa), chicken ovalbumin (44 kDa) and equine myoglobin (17 kDa).

Co-immunoprecipitation - N. benthamiana leaves were infiltrated with *Agrobacterium tumefaciens* strains (GV3101, MOG101) transformed with combinations of pBIN35S-*Nb*DBCP-4HA, pBIN35S-Rx-CC-4myc and pBIN35S-Rx1-4myc binary vectors and leaf material was harvested 2 days after infiltration. 100 mg of leaf material was ground in liquid nitrogen and resuspended in 1.5 mL extraction buffer (10% (v/v) glycerol, 25 mM Tris-HCl pH 7,5, 1 mM Na_2EDTA, 150 mM NaCl, 0.6 mg/mL Pefabloc SC, 20 mg/mL polyvinylpolypyrrolidone, 0.1 % (v/v) Tween 20, 5 mM DTT). The supernatant was passed through a 5 mL G25 Sephadex column after pelleting the cell debris. The resulting sample was incubated at 4°C with 50 μL magnetic beads (Miltenyi μMACs) for 2 hours. Unbound proteins were removed by washing 5 times with washing buffer (10% (v/v) glycerol, 25 mM Tris-HCl pH 7.5, 1 mM Na_2EDTA, 150 mM NaCl, 0.15% (v/v) Nonidet P-40, 5 mM DTT).

For Elution 1 the column was removed from the magnetic holder and moved to an Eppendorf tube. 45 μL of washing buffer was added to remove the magnetic beads from the column. Subsequently 15 μL of 4x NuPAGE LDS sample buffer (60 mM DTT) was added and the samples were incubated at 95°C for 5 minutes.

For Elution 2, 20 μL of 1x NuPAGE LDS sample buffer (95°C) was added directly to the column and incubated for 5 minutes. After 5 minutes the column was removed from the magnetic holder and moved to an Eppendorf tube. An additional 50 μL 1x NuPAGE LDS sample buffer (95°C) was added to the column to remove the beads and protein from the column.

Start material (before incubation with beads), the unbound fraction and the captured proteins were separated on NuPAGE novex 12% bis-tris gels in MES buffer (50 mM MES, 50 mM Tris base, 0.1% SDS, 1 mM EDTA pH 7.3) and blotted on polyvinylidene difluoride (PVDF) membranes for immunoblot analysis. Affinity-tagged proteins were detected using peroxidase conjugated antibodies (c-Myc: goat anti-c-Myc (Abcam 9132) and donkey anti-goat peroxidase conjugated (Jackson 705-035-147), HA: rat anti-HA HRP conjugated (Roche 12013819001)). Peroxidase activity was visualized with the SuperSignal™ West Dura and Femto Substrates (Thermo Scientific) and imaged in a Syngene G:BOX Chemi HR-16 Gel documentation system.

Fluorescence Anisotropy – Double-stranded DNA substrates with a concatenated GGATATCC site *Nb*Glk1-binding site (GGATATCC) was made by annealing synthetic oligonucleotides FA-5 and FA-6. FA-5 was end-labelled with FAM. Double-stranded DNA was annealed by mixing 10 μM concentrations of complementary oligonucleotides in 150 mM NaCl, 15 mM NaCitrate, heating to 95°C, and cooling to room temperature over 5 hours. Changes in anisotropy were measured using a Synergy™ H4 Fluorescence Spectrophotometer (BioTek) fitted with polarizing filters (λ_{em} = 528 nm, λ_{ex} = 485 nm, bandwidth = 20 nm, averaging time = 10 s). Anisotropy was determined using 10 nM fluorescein end-labelled oligonucleotides (Eurofins MWG) with variable protein in 20 mM Tris-HCl pH 7.4, 140 mM NaCl, 1 mM DTT. Anisotropy was calculated using Gen5 software (BioTek).

Laser Scanning Confocal Microscopy - Subcellular distribution studies were performed using a Zeiss LSM 510 confocal microscope (Carl Zeiss) and a X40 1.2 numerical aperture water-corrected objective as previously described (Slootweg et al., 2010). *A. tumefaciens* strain GV3101 (pMP90) transformed with GFP-fusion constructs of *Nb*DBCP wild type or E386L mutant variant were infiltrated on *N. benthamiana* leaves at final OD_{600} values of 0.5. Leaf epidermal cells transiently expressing these constructs were harvested between 48-72 dpi and imaged by confocal microscopy. For GFP and chlorophyll imaging, *N. benthamiana* epidermal cells were excited using an Argon laser at 488 nm and emissions were detected through 505-550 nm band-pass and 650 nm long-pass filters, respectively. A HeNe 543 nm laser-line was used to excite leaf samples for mCherry imaging and a 600-650 nm band-pass filter was used for detection. Confocal images were analysed using the Java application ImageJ (Abramoff et al., 2004).

Time Resolved FRET In Situ – A. tumefaciens strain GV3101 (pMP90) was transformed with constructs pK7WGF2 (GFP negative control), pK7WGF2-H2B (GFP-H2B positive control), pBIN35S-CP106, pBIN35S-Rx1, pK7WGF2-GFP-*Nb*DBCP (GFP-*Nb*DBCP) and experiments were performed as described (Fenyk et al., 2015).

N. benthamiana Hypersensitive Response Assay - *N. benthamiana* leaves were infiltrated with *A. tumefaciens* transformed with constructs pBIN35S-CP106, pBIN35S-Rx1, and pBIN35S-*Nb*DBCP-4HA at a $A_{600\,nm}$ = 0.1-0.5. Plants were incubated for 4 days at 25°C with 16 h of light. Leaves were harvested, visually inspected,

photographed and scored 1-5 for cell death as previously described (Townsend et al., 2017): 1 being no visual sign of any cell death whatsoever in the infiltrated region and 5 being complete cell death throughout the infiltrated region.

Virus Induced Gene Silencing (VIGS) - Three-week old *N. benthamiana* plants were co-infiltrated with *Agrobacterium* GV3101 strains for co-expression of TRV1 and TRV2 vectors. Silencing vectors used were pTRV2-GFP, pTRV2-SGT1, pTRV2-Δ*Nb*DBCP-1 and pTRV2-Δ*Nb*DBCP-2. Three or four weeks after inoculation, the upper leaves of the plant were used for transient expression and qPCR experiments to check for silencing levels using oligonucleotides F-q1 and R-q1.

VIGS Transient PVX Resistance Assay - The resistance mediated by Rx1 against PVX was assessed by co-expressing Rx1 with *Agrobacterium* GV3101 harbouring an amplicon of the PVX genome in *N. benthamiana* leaves. To observe either an increase or decrease in PVX resistance the Rx1 constructs were expressed at low levels using a leaky scan promoter (Slootweg et al., 2010) and relatively low densities of the *Agrobacterium* strain for agroinfiltration ($A_{600\ nm}$ = 0.1-0.05). Under these conditions Rx1 does not exhibit complete resistance and factors affecting Rx1 function are reflected in changes in PVX accumulation. *Agrobacterium* GV3101for expression of PVX (pPVX:GFP) was agroinfiltrated at $A_{600\ nm}$ = 0.002. At five days post-infiltration 3 x 6 mm diameter leaf discs were collected per infiltration spot and ground in 250 µL 50 mM phosphate buffer (pH 7) using a Tissuelyser II (Qiagen; 3 mm ball bearing, 2 x 1 min, 30 Hz). Cell debris was centrifuged for 10 min 5,000 g. 50 µL of the supernatant was used in a DAS-ELISA (Maki-Valkama et al., 2000) for PVX as described (Slootweg et al., 2010).

Expression Analysis by Quantitative Real-Time PCR - Total RNA was extracted from 50 mg leaf tissue using the Maxwell 16 simpleRNA extraction kit (Promega). cDNA synthesis was performed using the SuperScript III First-Strand Synthesis System (Invitrogen). Gene expression levels were analysed by qPCR in a reaction mix of 25 µl consisting of: 0.2 µM forward and reverse primers (each), 10 ng cDNA, 12.5 µl Absolute qPCR Sybergreen Fluorescein mix (ThermoFisher), and 12.5 µl MQ water. qPCR data was normalized against the actin reference gene. Relative expression levels were analysed by the comparative method ($2^{-\Delta\Delta Ct}$) using average threshold (Ct) values (Schmittgen and Livak, 2008).

Overexpression Transient Nb*Glk1-mediated PVX Resistance Assay - N. benthamiana* leaves were infiltrated with *Agrobacterium tumefaciens* transformed with pGr106 with and without constructs pBIN35S-*Nb*Glk1-4HA, and pBIN35S-*Nb*DBCP-4HA at a $A_{600\ nm}$ = 0.1-0.5. Leaves were grown for 4 days and then harvested. Three different 10 mm diameter leaf discs were excised and each disc was placed into a 96-well plate for each infiltrated area. The fluorescence intensity of each leaf disc was measured using a Synergy™ H4 Fluorescence Spectrophotometer (BioTek) (λ_{em} = 550 nm, λ_{ex} = 410 nm, bandwidth = 20 nm, averaging time = 10 s). An average of the fluorescence intensities for the three leaf discs was calculated to give a value for each infiltrated area. The fluorescence intensity of each averaged area was normalised to an infiltrated area on each leaf with only pGr106.

Overexpression Transient Rx1-mediated PVX Resistance Assay - N. benthamiana leaves were infiltrated with *A. tumefaciens* transformed with pGr208 with and without constructs for *Nb*DBCP or Rx1. To increase the probability of observing differences in PVX levels, a leaky scan Rx1 construct (with reduced translational efficiency) and relatively low $A_{600\ nm}$ value for *A. tumefaciens* transformed with pGr208 ($A_{600\ nm}$ = 0.002) were used. At 5 dpi, 4 x 3 mm leaf discs were collected from infiltrated zones, grounded in phosphate buffer (50 mM at pH 7) using the Tissue Lyzer (2 x 3 mm steel beads at 30 Hz for 30s) and used in a DAS-ELISA for PVX.

Structural Modelling - Protein fold searches using the Phyre^2 protein homology/analogy recognition engine version 2.0 (Kelley and Sternberg, 2009) were undertaken using amino acids 304-405 of *Nb*DBCP, using both normal and intensive modelling modes. The model was based on the crystal structure of the BPTF BD in complex with histone H4 acetylated at Lys16 (PDB accession number 3QZT) made using Chainsaw within the CCP4 package (Winn et al., 2011) and sequence alignments were generated by the Phyre^2 server. Side chain packing and energy minimization was performed using GalaxyRefine (Heo et al., 2013). Figures were generated using the PyMOL molecular graphics system (Schrodinger, 2010).

Phylogenetic Analysis – Protein sequences with a similar domain structure to *Nb*DBCP were identified using Simple Modular Architecture Research Tool (Letunic et al., 2015). Phylogenetic analysis was performed by Maximum Likelihood enacted in Phylogeny.fr (Dereeper et al., 2008).

Statistical Analysis - Error bars represent the standard error of the mean with the number of biologically independent replicates as indicated in the legend. Statistical comparisons (*p* values) were obtained from one-way ANOVA with the indicated post-hoc test unless otherwise indicated. *p* values in statistical comparisons are indicated in figures and indicate compared data sets as described in the figure legends.

Supplemental Table (Related to Supplemental Methods). The oligonucleotides used for this study.

**Primer Name**	**Orientation**	**Sequence**
*Nb*DBCP-1	Sense	5'-GGC CTC GAG GCC GTC GGA CAT ATT AAA G-3'
*Nb*DBCP-2	Antisense	5'-GGC CTC GAG TTA GGG CCC AGG TGA TGA ATG-3'
*Nb*DBCP-3	Sense	5'-GGC TCA TGA TCG AAA AAG AGT ACG GCA CAA C-3'
*Nb*DBCP-4	Antisense	5'-GGC GCG GCC GCC CTC CTT GAC CGT TTC TTT G-3'
*Nb*DBCP-5	Sense	5'-GGG GAC AAG TTT GTA CAA AAA AGC AGG CTA CAT GGA AAA AGA GTA CGG C-3'
*Nb*DBCP-6	Antisense	5'-GGG GAC CAC TTT GTA CAA GAA AGC TGG GTC ACC TCC TTG ACC GTT TCT TTG-3'
*Nb*DBCP-7	Sense	5'-GGC CTC GAG ATG ATC GAA AAA GAG TAC-3'
*Nb*DBCP-8	Antisense	5'-GGC CTC GAG TTA GGG CCC AGG TGA TGA ATG-3'
*Nb*DBCP-9	Sense	5'-CCC TAA GTC ATC CCC ACT GTC AAC TGC AGC TAA TG-3'
*Nb*DBCP-10	Antisense	5'-CAT TAG CTG CAG TTG ACA GTG GGG ATG ACT TAG GG-3'
*Nb*DBCP-11	Sense	5'-CAG AAA ACG GAC AAG TTC AAG AAT ATG ATC CGA C-3'
*Nb*DBCP-12	Antisense	5'-GTC GGA TCA TAT TCT TGA ACT TGT CCG TTT TCT G-3'
FA-5	Sense	5'-CGG ATA TCC ACG GAT ATC CAC GGA TAT CCA CGG ATA TCC ACG GAT ATC CC-3'
FA-6	Antisense	5'-GGG ATA TCC GTG GAT ATC CGT GGA TAT CCG TGG ATA TCC GTG GAT ATC CG-3'
F-q1	Sense	5'-CCG TCG AAG AGG AGT GTA GG-3'
R-q1	Antisense	5'-CCG TCG AAG AGG AGT GTA GG-3'

Figure S1 (Related to Figure 1). Maximum-likelihood phylogenetic tree showing the calculated relationship between higher plant species with a conserved SANT-type domain and BD structure. Numbers represent bootstrap values. Branch terminal labelling is in the format UniProt ID_Species name. *Nb*DBCP is boxed in red.

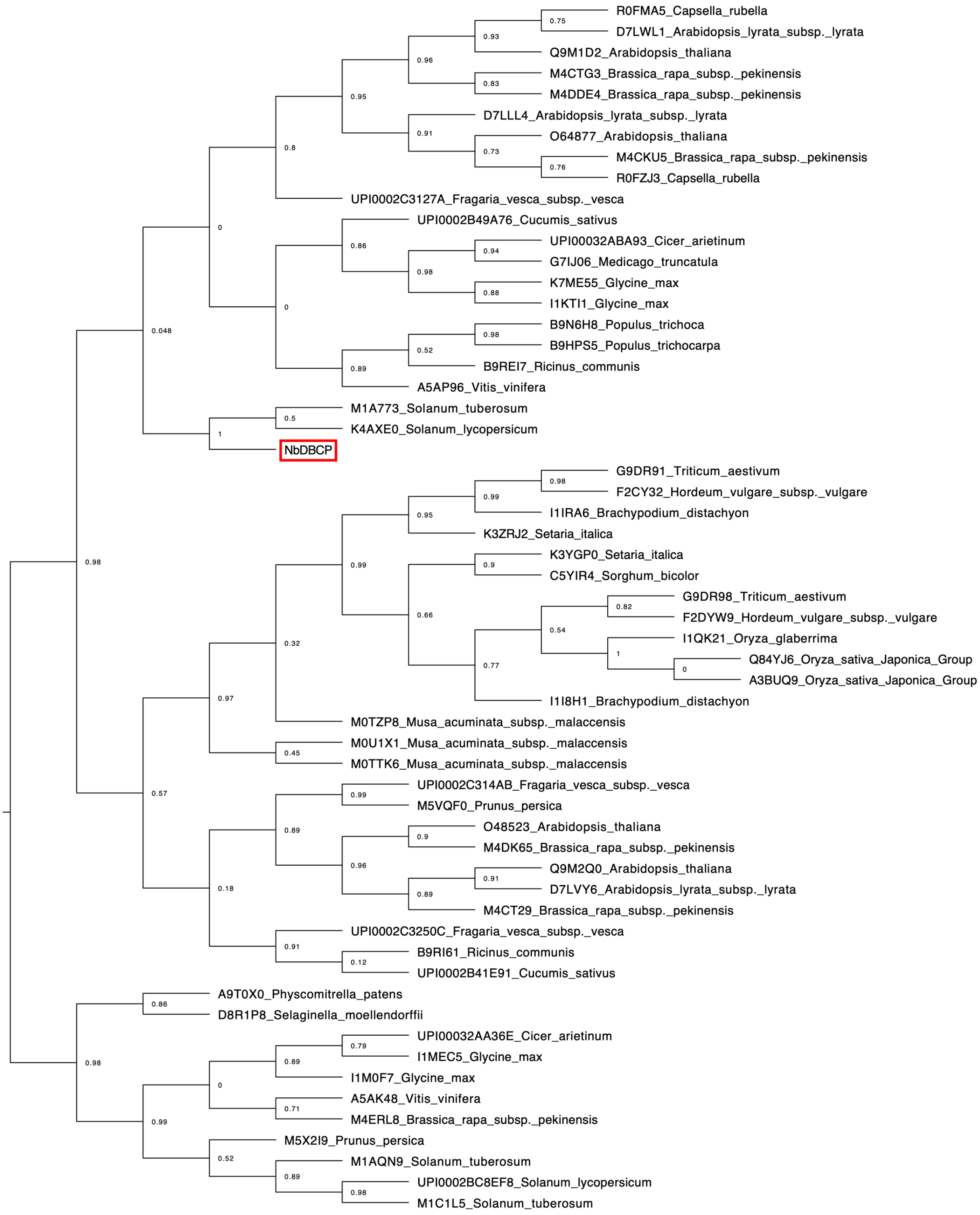

Figure S2 (Related to Figure 2). I_N/I_C ratio for Rx1-mCherry expressed with either GFP or *Nb*DBCP-GFP (mean ± 95% C.I).

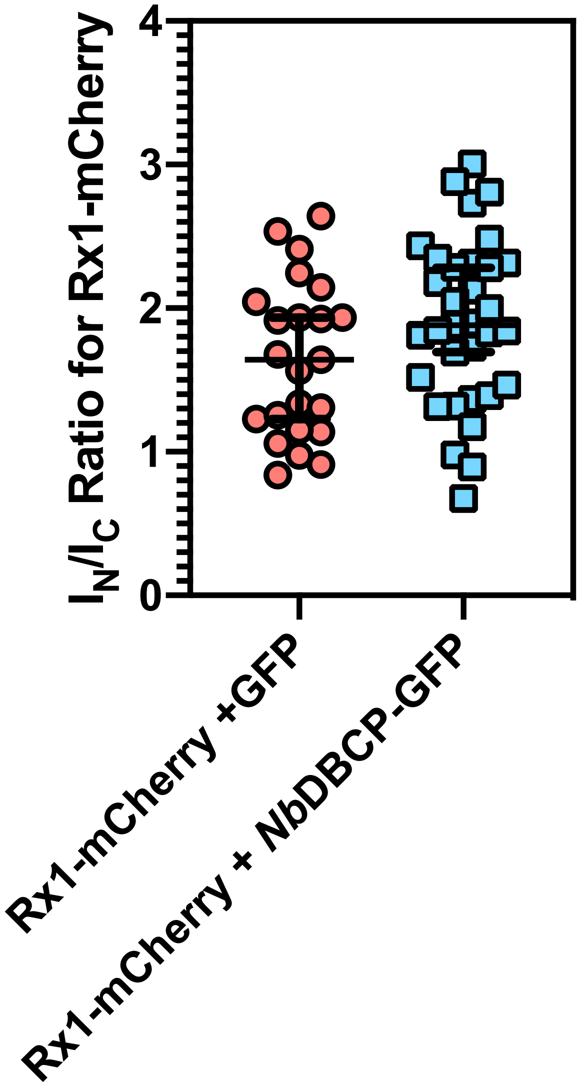

Figure S3 (Related to Figure 2). Subcellular distribution of *Nb*DBCP-GFP *in planta*. Representative overlay confocal images of *N. benthamiana* leaf epidermal cells transiently expressing *Nb*DBCP-GFP with or without P19, free GFP or an uninfiltrated leaf. Images were taken at 2 or 3 dpi. Scale bar = 10 µm. N = nucleus, n = nucleolus, SB = subnuclear bodies, C=cytoplasm, Ch = chloroplasts. **A.** Views of individual cells. **B.** View of a field of cells. White triangles point towards a cellular nucleus.

A.

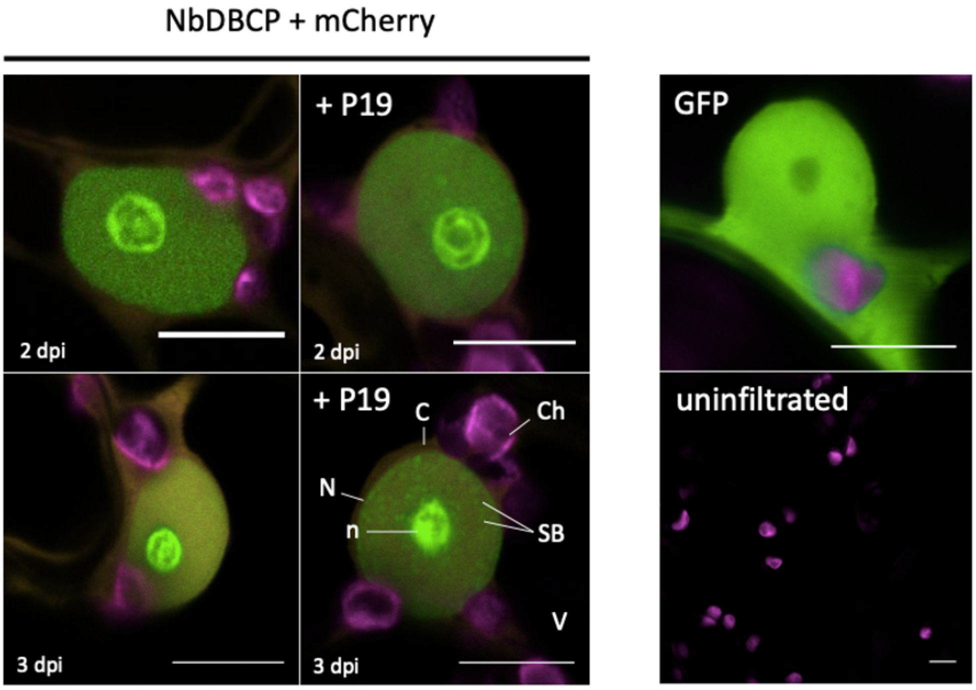

B.

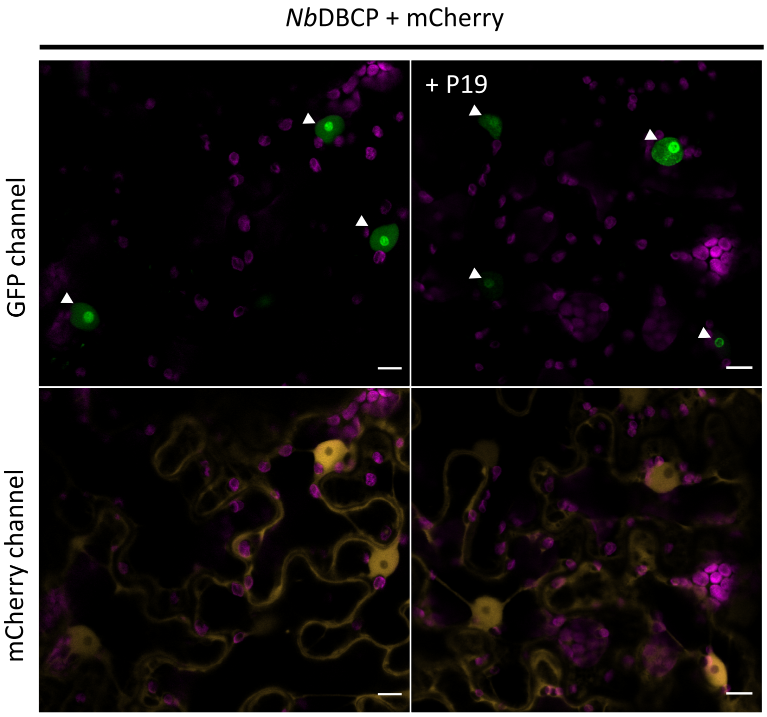

Figure S4 (Related to Figure 2). Immunoblot of mCherry constructs used for the confocal microscopy. Infiltrated *N. benthamiana* leaf samples were harvested at 3 dpi and protein from either total extract or the soluble fraction were used for used for Western blotting with an anti-mCherry antibody. The black triangle indicates the presence of a protein band with the expected size of Rx1-mCherry. CBB denotes Coomassie Blue stain loading control for the denoted samples.

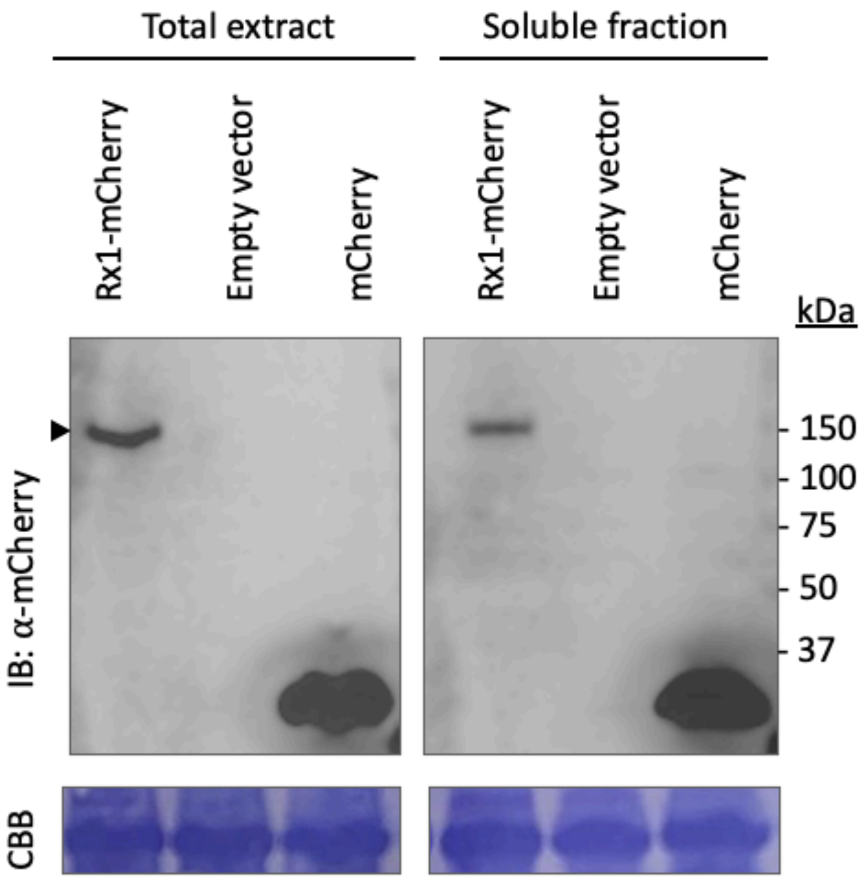

Figure S5 (Related to Figure 2). Co-expressing Rx1-mCherry with NbDBCP-GFP results in a significant increase in signal of the receptor in the nucleolus. Relative intensity ratios of Rx1-mCherry or free-mCherry in the nucleolus relative to the nucleoplasm ($I_{Nucleolus}/I_{Nucleoplasm}$) in the presence or absence of *Nb*DBCP-GFP. Crossbar represents the mean values. Data was taken from four independent experimental repeats and tested for normality using the Shapiro-Wilk test. Subsequently, statistical significance was calculated using the Wilcoxon Signed-Rank Test with $\alpha = 0.05$. Intensity of Rx1-mCherry in each compartment was determined using the ImageJ software. Signal from the vacuole was considered as background and subtracted from the signal of each compartment before taking a ratio.

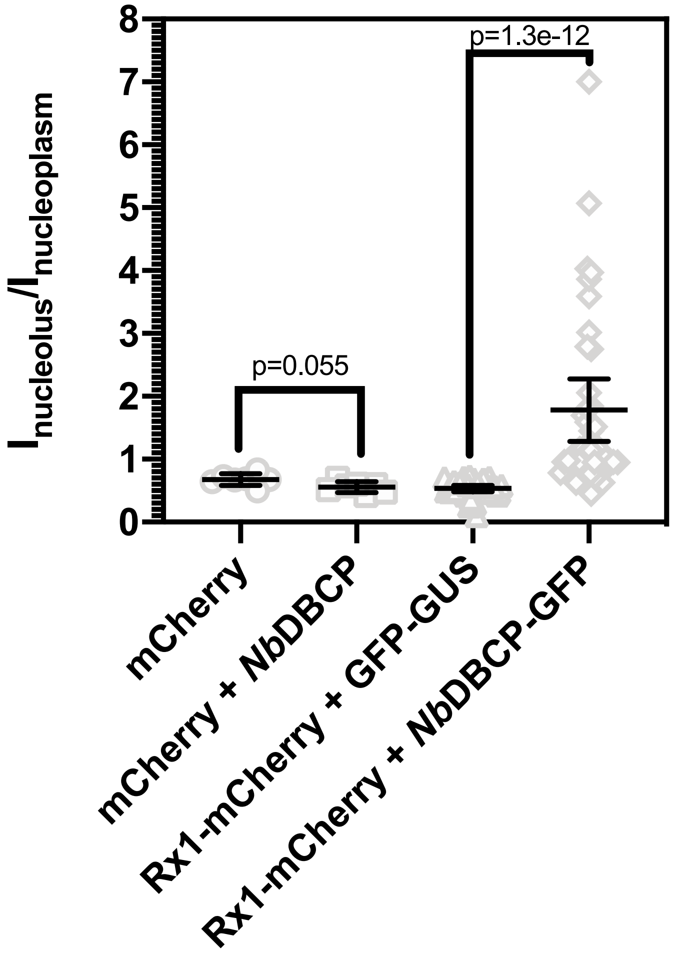

Figure S6 (Related to Figure 3). CP106 does not interact with *Nb*DBCP by Y2H. 1x1 Yeast Two-Hybrid screen of CP106(1-237) against amino acids 265-493 of *Nb*DBCP. CP106(1-237) was fused to the Gal4 DNA-binding domain and *Nb*DBCP(265-493) wild type and E386L was fused to the Gal4 activation domain. Plates were grown on medium lacking leucine and tryptophan (−L/−W) and medium lacking leucine, tryptophan, histidine (−L/−W/−H). **A**. Smad vs Smurf positive control **B**. Empty pB66 bait vs *Nb*DBCP(265-493) wild type in prey negative control. **C**. CP106(1-237) in pB66 bait plasmid vs empty pP7 prey negative control. **D**. CP106(1-237) in pB66 bait plasmid with *Nb*DBCP(265-493) wild type in pP7 prey plasmid.

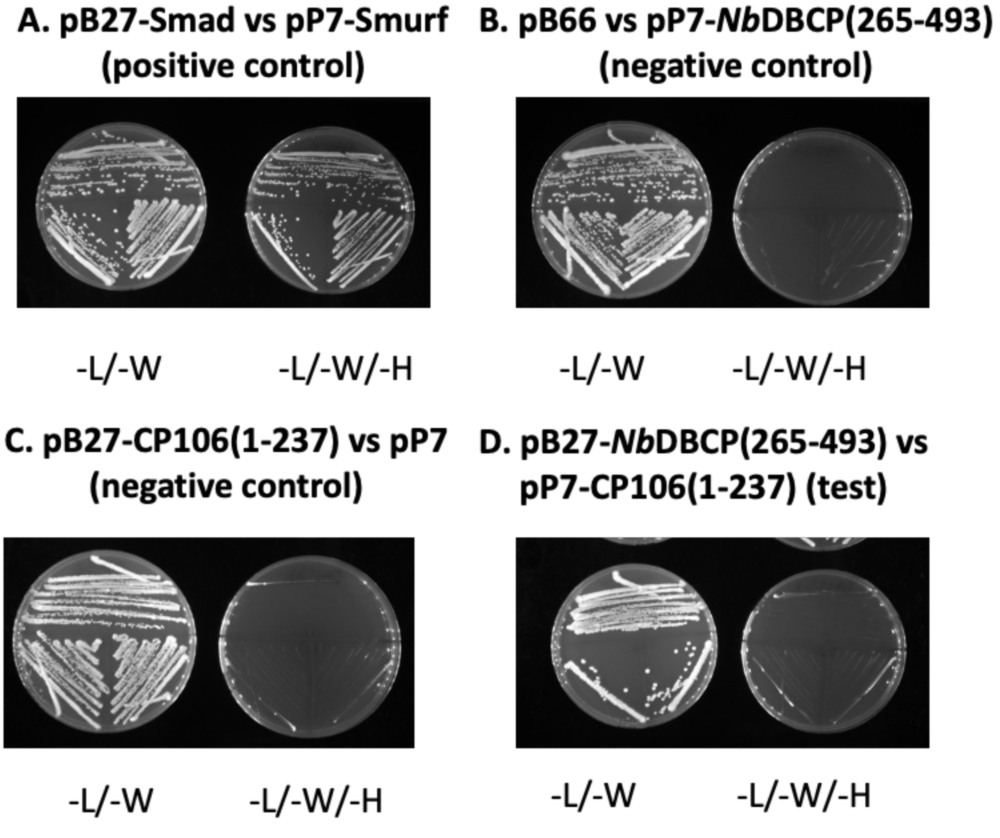

Figure S7 (Related to Figure 5). Representative photographs of *N. benthamiana* leaves infiltrated with pGR208 (that drives expression of a PVX amplicon) and/or full-length Rx1 in the presence/absence of *Nb*DBCP-WT or E386L variant. Images were taken at 5 dpi. Independent biological repeats are shown to demonstrate consistent results between leaf samples. Key: A1 = pGR208 + GFP; A2 = pGR208 + *Nb*DBCP -WT; A3 = pGR208 + *Nb*DBCP -E386L; B1 = pGR208 + Rx1 + GFP; B2 = pGR208 + Rx1 + *Nb*DBCP -WT; B3 = pGR208 + Rx1 + *Nb*DBCP -E386L.

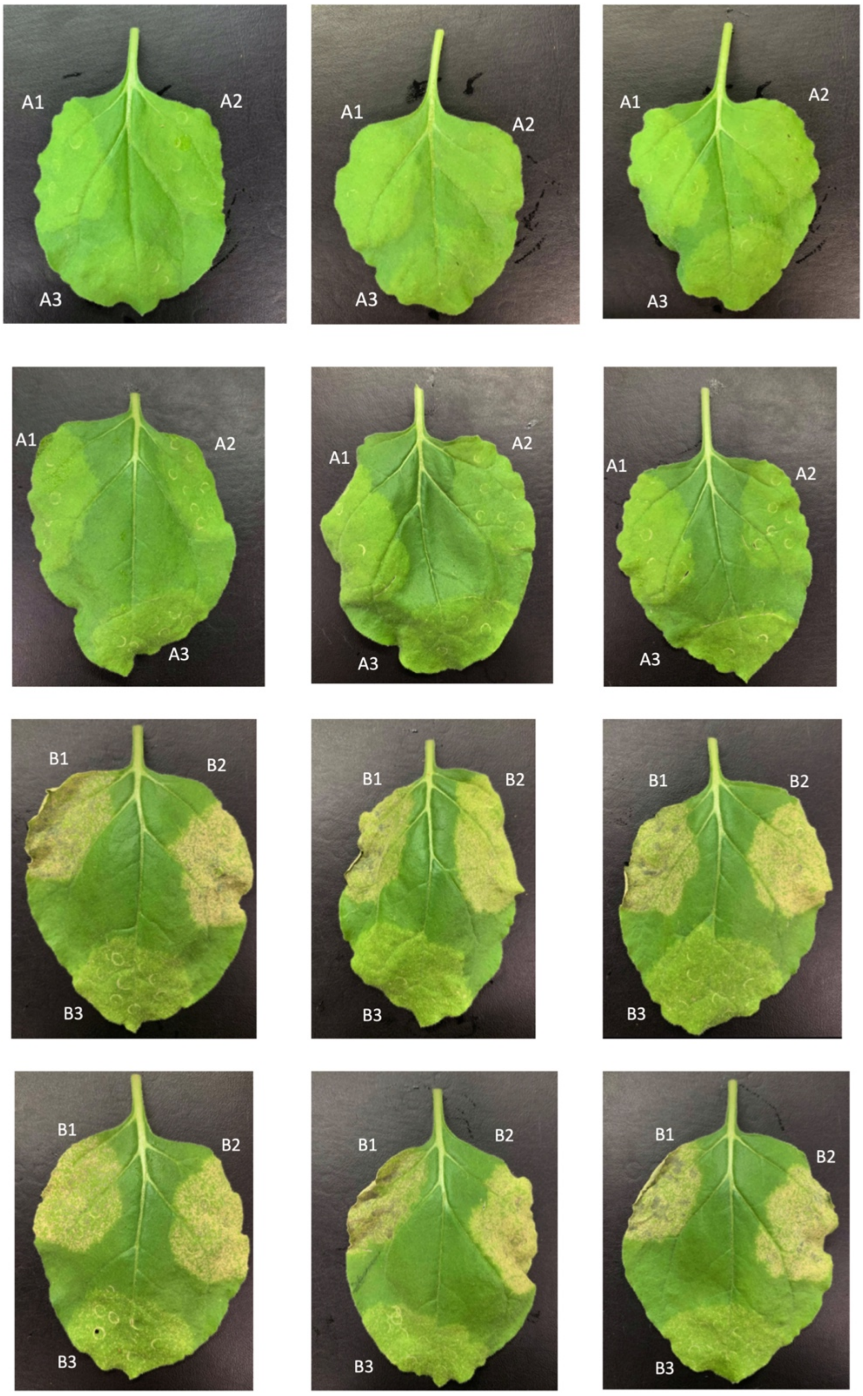

Figure S8 (Related to Figure 5). The N-terminus of the Rx1 NLR protein interacts with *Nb*DBCP wild type and E386L. 1x1 Yeast Two-Hybrid screen of Rx1(1-144) against amino acids 265-493 of *Nb*DBCP. Rx1(1-144) was fused to the Gal4 DNA-binding domain and *Nb*DBCP(265-493) wild type and E386L was fused to the Gal4 activation domain. Plates were grown on medium lacking leucine and tryptophan (−L/−W) and medium lacking leucine, tryptophan, histidine (−L/−W/−H), supplemented with 5, 10 or 20 mM 3-Amino-1,2,4-triazole (3AT). **A**. Smad vs Smurf positive control **B.** Empty pB27 bait vs *Nb*DBCP(265-493) wild type in prey negative control. **C.** Empty pB27 bait vs *Nb*DBCP(265-493) E386L in prey negative control. **D**. Rx1(1-144) containing bait vs empty pP7 prey negative control. **E.** Rx1(1-144) in pB27 bait plasmid with *Nb*DBCP(265-493) wild type in pP7 prey plasmid. **F.** Rx1(1-144) in pB27 bait plasmid with *Nb*DBCP(265-493) E386L in pP7 prey plasmid.

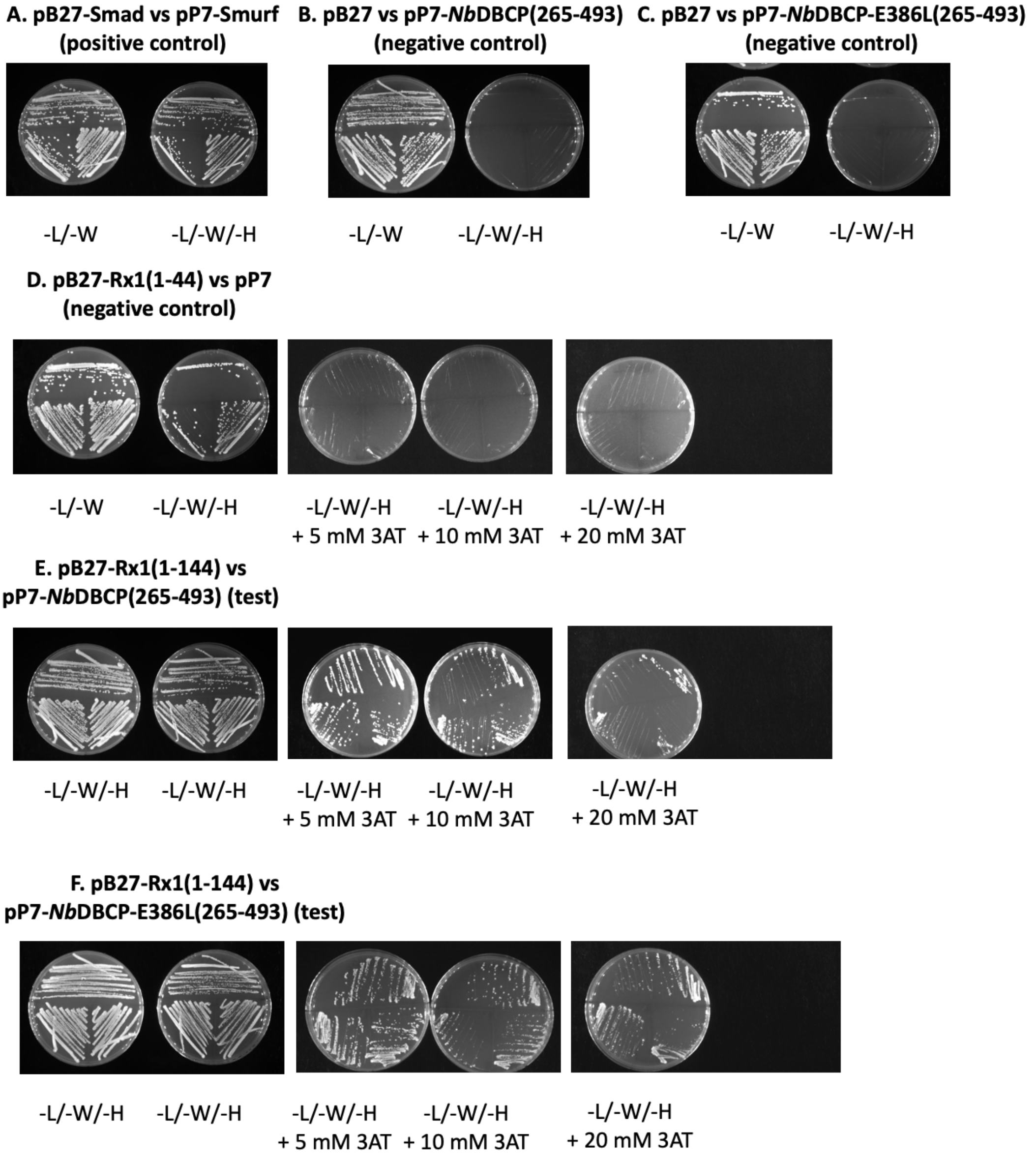

Figure S9 (Related to Figure 6). A). Immunoblotting of constructs used for the confocal imaging of *Nb*GLK1-GFP and *Nb*DBCP-HA. Total and soluble protein extracts from *N. benthamiana* leaves harvested at 2 dpi were used for Western blotting. Staining of membrane by Coomassie-Brilliant Blue (CBB) was performed as loading control. B). Subcellular localization patterns of *Nb*GLK1-GFP in the presence or absence of *Nb*DBCP-HA. Imaging was performed at 2 dpi by confocal microscopy of *N. benthamiana* epidermal cells expressing the appropriate construct combinations. White bar represents a scale of 10 µm. N = nucleus; n = nucleolus; V = vacuole; Ch = chloroplast.

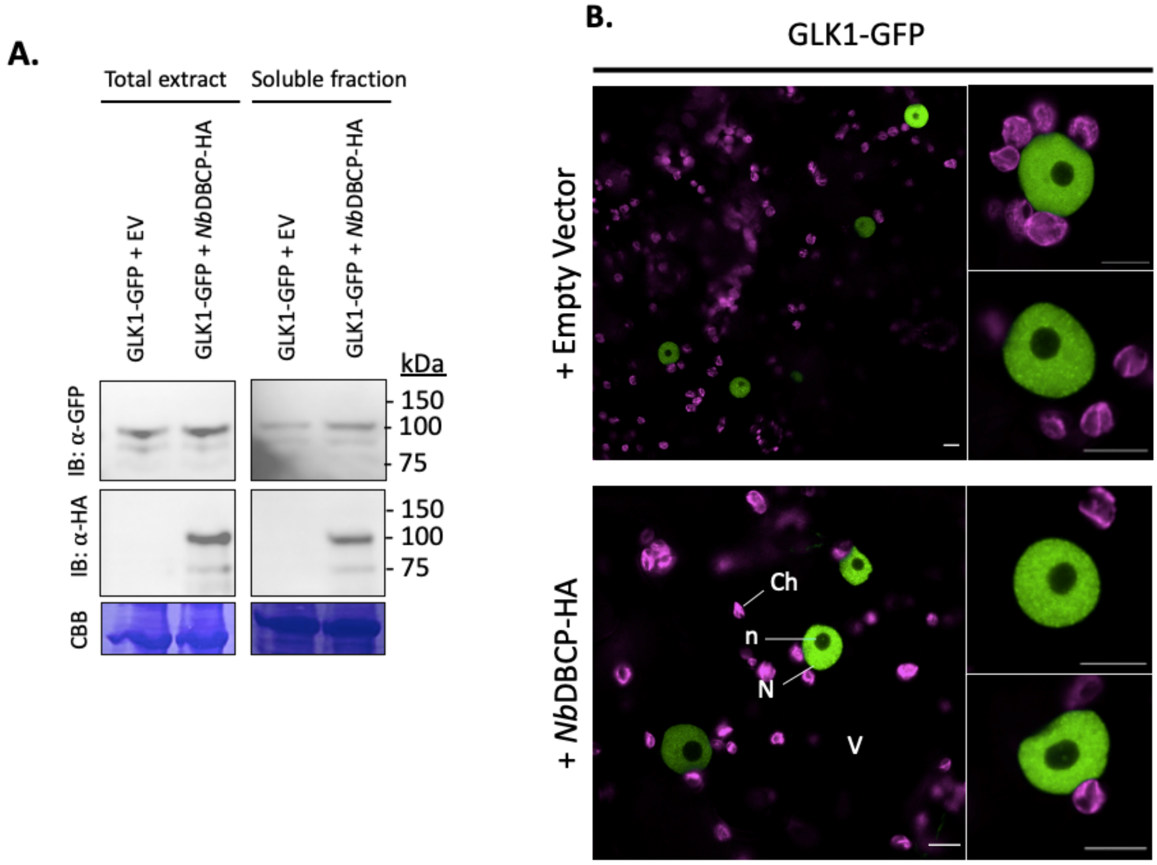

SUPPLEMENTAL REFERENCES

Abramoff, M.D., Magalhaes, P.J., Ram, S.J. (2004). Image Processing with ImageJ. Biophotonics Int 11:36-42.

Dereeper, A., Guignon, V., Blanc, G., Audic, S., Buffet, S., Chevenet, F., Dufayard, J.F., Guindon, S., Lefort, V., Lescot, M., et al. (2008). Phylogeny.fr: robust phylogenetic analysis for the non-specialist. Nucleic Acids Res 36:W465-469.

Fenyk, S., Townsend, P.D., Dixon, C.H., Spies, G.B., de San Eustaquio Campillo, A., Slootweg, E.J., Westerhof, L.B., Gawehns, F.K., Knight, M.R., Sharples, G.J., et al. (2015). The Potato Nucleotide-binding Leucine-rich Repeat (NLR) Immune Receptor Rx1 Is a Pathogen-dependent DNA-deforming Protein. J Biol Chem 290:24945-24960.

Fromont-Racine, M., Rain, J.C., and Legrain, P. (1997). Toward a functional analysis of the yeast genome through exhaustive two-hybrid screens. Nat Genet 16:277-282.

Gabriels, S.H., Takken, F.L., Vossen, J.H., de Jong, C.F., Liu, Q., Turk, S.C., Wachowski, L.K., Peters, J., Witsenboer, H.M., de Wit, P.J., et al. (2006). CDNA-AFLP combined with functional analysis reveals novel genes involved in the hypersensitive response. Mol Plant Microbe Interact 19:567-576.

Gabriels, S.H., Vossen, J.H., Ekengren, S.K., van Ooijen, G., Abd-El-Haliem, A.M., van den Berg, G.C., Rainey, D.Y., Martin, G.B., Takken, F.L., de Wit, P.J., et al. (2007). An NB-LRR protein required for HR signalling mediated by both extra- and intracellular resistance proteins. Plant J 50:14-28.

Haseloff, J., Siemering, K.R., Prasher, D.C., and Hodge, S. (1997). Removal of a cryptic intron and subcellular localization of green fluorescent protein are required to mark transgenic Arabidopsis plants brightly. Proc Natl Acad Sci U S A 94:2122-2127.

Karimi, M., Inze, D., and Depicker, A. (2002). GATEWAY vectors for Agrobacterium-mediated plant transformation. Trends Plant Sci 7:193-195.

Kelley, L.A., and Sternberg, M.J. (2009). Protein structure prediction on the Web: a case study using the Phyre server. Nat Protoc 4:363-371.

Letunic, I., Doerks, T., and Bork, P. (2015). SMART: recent updates, new developments and status in 2015. Nucleic Acids Res 43:D257-260.

Liu, Y., Schiff, M. and Dinesh-Kumar, S.P. (2002). Virus-induced gene silencing in tomato. Plant J 31:777-786.

Maki-Valkama, T., Valkonen, J.P., Kreuze, J.F., and Pehu, E. (2000). Transgenic resistance to PVY(O) associated with post-transcriptional silencing of P1 transgene is overcome by PVY(N) strains that carry highly homologous P1 sequences and recover transgene expression at infection. Mol Plant Microbe Interact 13:366-373.

Schmittgen, T.D., and Livak, K.J. (2008). Analyzing real-time PCR data by the comparative C(T) method. Nat Protoc 3:1101-1108.

Schrodinger, LLC. (2010). The PyMOL Molecular Graphics System, Version 1.3r1.

Slootweg, E., Roosien, J., Spiridon, L.N., Petrescu, A.J., Tameling, W., Joosten, M., Pomp, R., van Schaik, C., Dees, R., Borst, J.W., et al. (2010). Nucleocytoplasmic distribution is required for activation of resistance by the potato NB-LRR receptor Rx1 and is balanced by its functional domains. Plant Cell 22:4195-4215.

Townsend, P.D., Dixon, C.H., Slootweg, E.J., Sukarta, O.C., Yang, A.W., Hughes, T.R., Sharples, G.J., Palsson, L.O., Takken, F.L.W., Goverse, A., et al. (2017). The intracellular immune receptor Rx1 regulates the DNA-binding activity of a Golden2-like transcription factor. J Biol Chem.

Winn, M.D., Ballard, C.C., Cowtan, K.D., Dodson, E.J., Emsley, P., Evans, P.R., Keegan, R.M., Krissinel, E.B., Leslie, A.G., McCoy, A., et al. (2011). Overview of the CCP4 suite and current developments. Acta Crystallogr D Biol Crystallogr 67:235-242.
